# Aptamers against Immunoglobulins: Design, Selection and Bioanalytical Applications

**DOI:** 10.3390/ijms21165748

**Published:** 2020-08-11

**Authors:** Zsófia Bognár, Róbert E. Gyurcsányi

**Affiliations:** BME “Lendület” Chemical Nanosensors Research Group, Department of Inorganic and Analytical Chemistry, Budapest University of Technology and Economics, Szent Gellért tér 4, H-1111 Budapest, Hungary; zsofia.bognar@mail.bme.hu

**Keywords:** aptamer, antibody, immunoglobulin, diagnostic, probe design

## Abstract

Nucleic acid aptamers show clear promise as diagnostic reagents, as highly specific strands were reported against a large variety of biomarkers. They have appealing benefits in terms of reproducible generation by chemical synthesis, controlled modification with labels and functionalities providing versatile means for detection and oriented immobilization, as along with high biochemical and temperature resistance. Aptamers against immunoglobulin targets—IgA, IgM, IgG and IgE—have a clear niche for diagnostic applications, therefore numerous aptamers have been selected and used in combination with a variety of detection techniques. The aim of this review is to overview and evaluate aptamers selected for the recognition of antibodies, in terms of their design, analytical properties and diagnostic applications. Aptamer candidates showed convincing performance among others to identify stress and upper respiratory tract infection through SIgA detection, for cancer cell recognition using membrane bound IgM, to detect and treat hemolytic transfusion reactions, autoimmune diseases with IgG and detection of IgE for allergy diseases. However, in general, their use still lags significantly behind what their claimed benefits and the plethora of application opportunities would forecast.

## 1. Introduction

**Figure 1:**
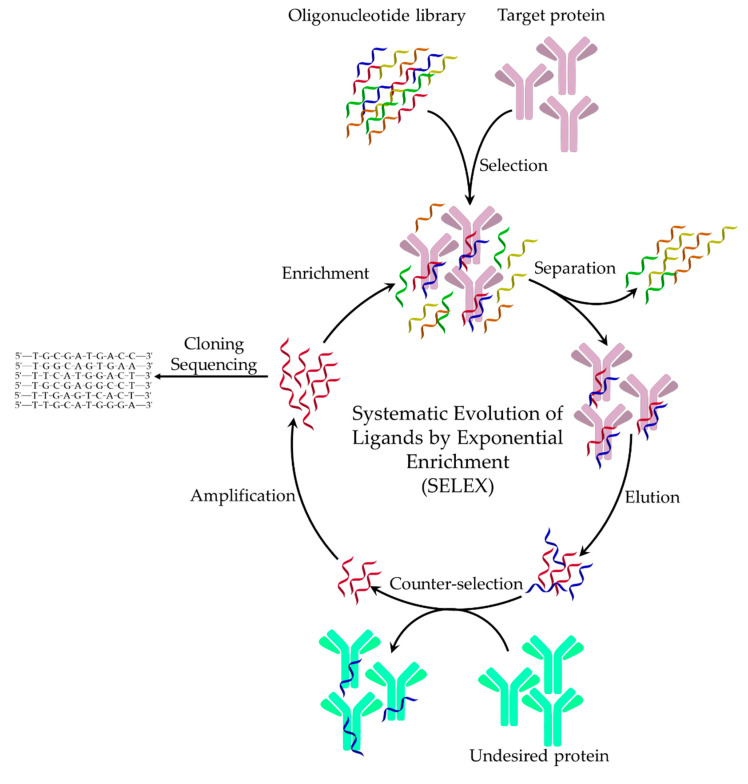
Schematic illustration of the SELEX procedure.

The expression of aptamer was first coined 30 years ago by Ellington and Szostak for RNA sequences that bind specifically to organic dye molecules. It is based on the Latin term “*aptus*” meaning to fit, which refers to the binding between the selected RNA strand and the target molecule [1]. At the same time, Tuerk and Gold introduced the expression of Systematic Evolution of Ligands by Exponential Enrichment (SELEX) for production of high-affinity RNA ligands to bacteriophage T4 DNA polymerase [2]. This is an in vitro process that aims at isolation and amplification of the nucleic acid candidates with highest specificity and affinity from a nucleic acid library comprising a high number of different sequence strands with the same length. To enable polymerase chain reaction (PCR)-based amplification (or in vitro transcription), the variable randomized sequence of each oligonucleotide, typically 30–50 nt, is flanked by two ca. 18–30 nt fixed sequence regions for primer binding. In the selection step, a library of oligonucleotides consisting of up to ca. 10^15^ different sequences is incubated with the target molecule with the expectation that some of the oligonucleotides will have the right sequence to fold in structures enabling their binding to the target. After incubation, the unbound sequences are separated, followed by a more stringent elution and the recovery of the target bound oligonucleotides, i.e., putative aptamers. These sequences are then PCR amplified and the enriched pool is used in the subsequent round of the selection along with the original library. These steps are repeated 5–15 times to enrich the aptamer candidates with high binding affinity to the target. These are then cloned, sequenced and characterized in terms of binding affinity and kinetics as well as selectivity against typical interferents or the sample matrix. The target selectivity of the enriched pool can be increased by counter-selection (counter-SELEX) [3]. During counter-selection, the library is screened for critical interferents, (e.g., close homologs of the target, or structurally-similar compounds); however, contrary to SELEX, the non-bound sequences are recovered and the bound are discarded. The selectivity of the resulting aptamers can be increased by including such counter-selection steps in the SELEX and using further only sequences that do not bound to the interferents (Figure 1). This is extremely important when generating aptamers for diagnostic applications as their cross-reactivity can be effectively minimized. Of note, the counter-selection can be made against the sample matrix itself, e.g., by using magnetic nanoparticles with autoreactive cyanuric-groups on their surface that by incubation in target-free serum are modified with typical serum proteins [4]. Aptamers, which can bind universally on Fc region of antibodies can be selected with target replacement strategy coupled to conventional SELEX method, i.e., to achieve universal binding capability, multiple immunoglobulin subclasses have to be consecutively substituted in a single SELEX process [5].

Several different SELEX methods have been used to generate immunoglobulin aptamers including homogeneous, heterogeneous, bead-based SELEX processes, CE-SELEX (capillary electrophoresis—SELEX), µFFE-SELEX (micro-free flow electrophoresis—SELEX) and fully integrated selection processes on microfluidic devices (M-SELEX). For aptamer selection against rIgG [6] and IgE [7], homogenous processes were used and the separation of bound and unbound nucleic acids was achieved by nitrocellulose filtration. On the surface of the nitrocellulose membrane, the protein targets were adsorbed and therefore the strongly bound nucleic acids are retained and could be amplified. However, non-specific adsorption might interfere with the selection, thereby a negative (counter) selection step was implemented against the nitrocellulose membrane to eliminate sequences with affinity for the nitrocellulose. In addition, the process might take up to several weeks to gain high affinity aptamers. Dramatically faster homogeneous aptamer selections can be made by electrophoretic methods that are inherently well suited to separate free- and protein target-bound sequences based on the significant difference in their electrophoretic mobility [8]. Using the CE-SELEX method, Bowser et al. [9] showed that high affinity aptamers can be selected for IgE in only two rounds of selection along with shortening the selection procedure down to two days. However, to achieve optimal resolution CE methods generally allow only for very small sample injections (a few nL) that is problematic in terms of accommodating the full library of sequences, unless very high concentrations are used. This problem can be circumvented by µFFE during which a continuous pressure-driven flow of library-target mixture is deviated laterally from the direction of the flow by a perpendicularly applied electrical field. Thus, the separation is based on the electrophoretic mobility difference of the components, as in classical CE, which enables a ca. 300-fold improvement in DNA library size, large sample sizes and much smaller applied voltages. µFFE could be used for continuous introduction, separation and collection of IgE aptamers, in practically one round, with similar affinities as by CE and conventional SELEX [10].

Heterogeneous SELEX methods are reliant on the immobilization of the target antibody on various solid carriers, e.g., surface of microtiter plate wells or PCR tube [5,11,12,13,14,15]. The immobilization of the target enables simple and fast separation of strongly and weakly bound nucleic acid sequences. However, negative selection against the blocked carrier surface is required to eliminate the non-specific binding sequences and relatively large amounts of target is required. Using magnetic beads for target immobilization, which is one of the most popular methods, the amount of the target can be reduced, the library-target reaction is performed in the solution bulk that avoids mass transfer limitations of the planar carriers, and the separation can be conveniently performed by applying a magnetic field. Moreover, magnetic nanoparticles with a large number of surface functionalities are available to facilitate the immobilization of the target IgGs, e.g., by coupling carboxylated beads with amino groups of the antibody [16], through His-tags [17] and oriented immobilization via Protein A or Protein G [18,19,20] for selection of Fab specific aptamers. Magnetic separations can be integrated in microfluidics and combined with SELEX (M-SELEX) to ensure an efficient, compact and fast screening of aptamers against the target. These systems were evaluated for the selection of as SIgA [21] and IgE [22,23,24] selective aptamers. Generally, such microfluidic devices comprise two chambers: a selection chamber and an amplification chamber. The nucleic acid library is introduced into the selection chamber where interaction with target functionalized magnetic beads happens. The loosely bound nucleic acids are washed out from the chamber, followed by the elution of the tightly bound aptamer candidates, which are transferred electrophoretically to the amplification chamber, where the aptamer candidates are amplified with PCR. The enriched pool is transferred back to the selection chamber for further affinity selection.

Aptamer selection against membrane bound antibodies (mIgM) were based on cell-SELEX technique, i.e. live cells were used as targets for selection [25]. The cell-SELEX has clear advantages in terms of circumventing the separation/purification of the target IgM and ensuring the native conformation of the target antibodies. To further increase the aptamer selectivity, so-called ligand-guided selection (LIGS) can be used [26]. This method employs a natural, highly specific antibody to the targeted epitope, which is used to outcompete and elute specific aptamers binding to the same epitope from an enriched nucleic acid pool, which are then collected and amplified.

These concepts proved to be successful to generate also DNA aptamers, although DNA strands were initially thought to be less prone than RNA to fold into strong secondary structures/functional motifs thought to be essential for high binding affinity aptamers.

**Figure 2:**
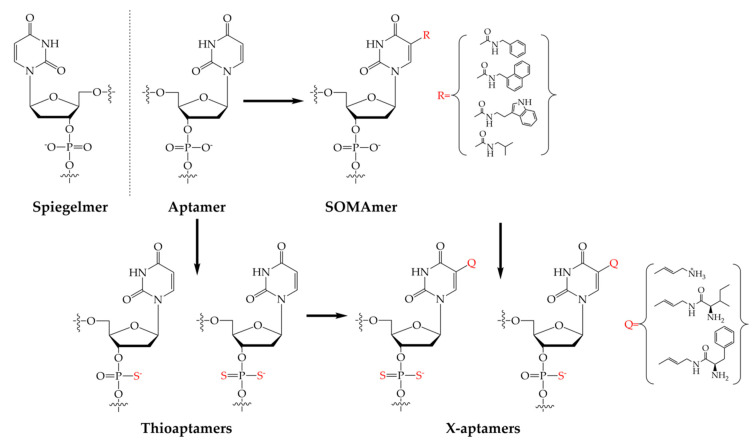
The most widely used modified aptamer derivatives: Spiegelmers, SOMAmers, Thioaptamers and X-aptamers. R and Q are referring to ligands that can be coupled to the bases to increase the affinity of the aptamer for the target.

During the last three decades, the selection methodology as well as implementation of non-natural nucleotides-modified nucleic acid aptamers went through tremendous progress to ensure the selection of aptamers with proper binding properties. To achieve higher binding affinity and inhibit digestion by cellular nucleases, various modifications were implemented that targeted the terminus of the strands, thesugar rings and bases, including the insertion phosphodiester linkages or producing the mirror-image of the D-nucleic acid strand. The most commonly used non-natural aptamers are Spiegelmers, SOMAmers, thioaptamers and X-aptamers (Figure 2). Spiegelmers are built up from non-natural L-nucleotides exhibiting nuclease resistance. Spiegelmers are also not recognized by polymerase enzymes during the amplification steps of SELEX and therefore cannot be obtained directly but by using mirrored targets and the natural D-nucleotides [27]. In SOMAmers (Slow Off-rate Modified Aptamers), chemically-modified nucleotides to infer protein-like properties are synthetized to achieve low dissociation constant. This is done through modification of the dU bases of the aptamers at the 5′ position with hydrophobic moieties, which improves their binding affinity, kinetics and selectivity and also increases their resistance to enzymatic degradation. In contrary to the Spiegelmers, SOMAmers can be selected and amplified using the conventional SELEX method with the modified nucleotides [28]. Thioaptamers are synthetized to accomplish stable cellular nucleic acid strands with the substitution of non-bridging phosphoryl oxygen atoms with sulfur. Similar to SOMAmers, they can be amplified by PCR, however the sulfur substitution can increase the non-specific interactions [29,30]. Using click-chemistry and phoshporothioate backbone a wide variety of chemically-modified aptamers can be selected with increased binding affinity and nuclease resistivity. The 5′ position of the aptamer was modified with drug-like molecules, resulting in the next generation of the aptamers: X-aptamers, aptamers that include X-modified bases [31,32].

In principle, aptamers have essential advantages over antibodies. This includes the purely chemical synthesis that ensures practically no batch-to-batch variations, the fact that the selection can be conveniently restricted to a targeted epitope and the excellent thermal and chemical resistance (as well as biochemical in the case of non-natural aptamers). Moreover, various modifications can be implemented without activity loss, which allows the application of aptamers on many detection platforms. However, antibodies are not only competitors of aptamers as diagnostic reagents but also targets for aptamer selection. The purpose of this review is to compile and evaluate aptamers selected for the recognition of antibodies—IgA, IgM, IgG and IgE—as well as their design, properties and diagnostic applications. Such a review is motivated also by the present context of COVID-19 pandemics, in which the indirect virus detection is largely based on the detection of IgM and IgG produced by the immune system against the virus.

## 2. Antibodies as Diagnostic Biomarkers

Since the production, overproduction or the absence of antibodies are highly correlated with several diseases and infections, they are frequently targeted diagnostic biomarkers. Detection of Fab domain ensures high specificity and the detection of the Fc domain provide generality to their detection. There are five subgroups of immunoglobulins (Igs): IgM, IgG, IgA, IgD and IgE (Table 1). As a primary immune response, IgM antibodies are secreted, therefore they can be useful targets for acute exposure diagnosis. However, during infection switching occurs to other subgroups, most importantly to IgG that is responsible for the neutralizing immune response and has the highest concentration and longest half-life in the blood. IgA is found on the mucosal surface with direct neutralization of toxins, bacteria and viruses. IgD is a membrane bound antibody and plays a major role in cell activation regulatory, however its processes are not totally understood. IgE is related to allergic reactions, although its concentration is negligible compared to the IgG, thereby its diagnostic application might be difficult [33].

As biomarkers, antibodies are mainly used for the diagnosis of various infections, autoimmune disease, allergy, immune deficiencies and cancer [34]. Upon infection, the B cells are stimulated by the antigen and the production of antigen specific antibodies starts. Firstly, IgM is secreted during the primary immune response in the first 4–96 h, whereas IgG and IgA antibodies are produced 7–10 days after infection [35]. Therefore, the type of the antibody is indicative of the elapsed time from infection, e.g., alleviating discrimination the acute or chronic stage. Moreover, the antigen specific Fab domain defines the specificity of the antibody. These features are the core of the point-of-care (PoC) tests for COVID-19, where the IgM and IgG concentration from fingertip or venous blood is determined by lateral flow assays or ELISA (enzyme-linked immunosorbent assay) methods [36].

During autoimmune diseases, IgG and IgM autoantibodies are produced as loss of self-tolerance against self-antigens. There are environmental and inherited factors contributing to autoimmune disorders. In the case of Sjorgen’s syndrome, rheumatoid arthritis, Alzheimer’s disease and cancer, autoantibodies can be early stage biomarkers, since they can be detected many years before the expression of symptoms. Based on the specificity and the concentration of autoantibodies prognosis, disease staging and treatment monitoring can be performed [33].

Allergic diseases are caused by excessive sensitivity of the immune system to otherwise largely harmless agents, e.g. pollen and food ingredients. The exposure to allergens triggers the production of antigen specific IgE and the highly elevated levels are indicative of the allergic reaction [37,38].

The absence of normal immunoglobulin concentration can indicate immunodeficiency, which arises from a defect in IgG, IgM and IgA production. Immune deficiency is diagnosed by measuring serum immunoglobulin level before and after immunization with pneumococcus and influenza vaccine. If the antibody concentration is not raised upon immunization, antibody deficiency has occurred, indicating the disability of the body to eliminate pathogens, which might cause repetitive infection and organ damages [39,40].

The immune system is able to sense extraordinary expression of cellular components associated with cancer evolution. The secreted antibody is specific for the components eliminated by the tumor cell, thereby the own immune response of the patent can be used to detect cancer at an early stage [41].

**Table 1:** Types of immunoglobulins with their structure, some associated diseases and clinically relevant concentration.

Type	Structure	Disease	Body Fluid	Normal Concentration	Altered Concentration	Ref.
**IgA**	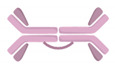 monomer, dimer	Psychological stress	saliva	148.2 µg/mL	243.2 µg/mL (intensified training)	[42]
					316.9 µg/mL (competition)	
		chronic hepatitis B	serum	2.27 mg/mL	2.50 mg/mL	[43]
		HIV-1		1.27 mg/mL	2.67 mg/mL	[44]
**IgM**	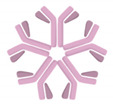 pentamer, monomer (membrane bound)	sickle cell disease (SC)	serum	1.11 mg/mL	2.87 mg/mL	[45]
		chronic hepatitis B		0.92 mg/mL	1.38 mg/mL	[43]
		HIV-1		1.39 mg/mL	1.52 mg/mL	[44]
**IgG**	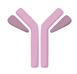 monomer	chronic hepatitis B	serum	11.44 mg/mL	16.83 mg/mL	[43]
		HIV-1		12.8 mg/mL	20.2 mg/mL	[44]
		ulcerative colitis		10.3 mg/mL	11.1 mg/mL	[46]
**IgE ^1^**	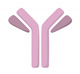 monomer	non-allergic asthma	serum	0.212 µg/mL	1.025 µg/mL	[47]
		allergic asthma			4.120 µg/mL	
		atherosclerosis		0.100 µg/mL	2.63 µg/mL	[48]
		psoriasis		0.113 µg/mL	0.574 µg/mL	[49]
**IgD**	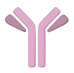 monomer	alcoholism	serum	20.2 mg/mL	27.3 mg/mL	[50]

^1^ Concentrations were calculated with 1 IU = 2.42 ng expression [51].

## 3. IgA Detection with Aptamers

Salivary secretory immunoglobulin A (SIgA) and salivary α-amylase (sAA) are immunological stress markers and increasing concentrations may enable the discrimination between comfortable and uncomfortable physical states. Moreover, SIgA is an indicator of upper respiratory tract infection. While there are simple, commercialized platforms to measure the sAA concentration based on the amylase activity [52], the results may be highly influenced by the salt concentration and the pH. Thereby, for proper physical state determination, the measurement of SIgA is necessary. The use of aptamers for the selective recognition of SIgA is rendered difficult by the fact that, since SIgA is hydrophilic and negatively charged owing to the dominance of hydrophilic amino acids in the Fc domain, there is an electrostatic repulsion between putative aptamers and SIgA [53].

A microfluidic-based aptamer selection was developed with integrated selection and amplification method for aptamer selection to IgA as representative target. The device was built up with two chambers for selection and amplification connected with a serpentine-shaped microchannel. Magnetic beads were modified with IgA placed into the selection chamber, where the strongly bound aptamers were transferred by thermal elution to the amplification chamber, to perform PCR amplification directly on the surface of primer-modified magnetic beads. After PCR amplification, the nucleic acid strands were reintroduced in the separation chamber for further affinity-based selection for the target. Upon completion of the SELEX procedure off-chip amplification of the aptamer candidates and the original DNA pool was performed using fluorescent forward primer. Affinity characterization by fluorescent binding assay was accomplished on IgA-modified magnetic beads, where the aptamer candidates showed significant increase in fluorescent intensity compared to the original DNA library. The results suggest that this magnetic bead-based multiround SELEX process on a microchip has the capability for aptamer selection for antibodies within 4 h [21]. However, the aptamer candidates did not reach desired binding affinity for immunoanalytical assays, most likely due to the electrostatic penalty in the interaction. This fundamental limitation could be circumvented with modified aptamers with enhanced hydrophobic interaction properties, which was published by Minagawa et al. [16]. Using the base-appended base (BAB) approach, where U^gu^ bases (U^gu^ stands for a guanine base at the 5th position of the uracil) were used to increase the binding strength of the fluorescent-labeled aptamer–target interaction as determined by fluorescence polarization in diluted human saliva. The selected aptamer (IgA^gu1^) was bound to the SIgA with high specificity and showed no cross-reactivity to IgG and serum IgA (Table 2). The sensitivity of fluorescence polarization detection can be increased by reducing the molecular weight of the fluorescent-labeled aptamer. Therefore, the length of the aptamer has been reduced in two steps (IgA^gu1-3^ and IgA^gu1-3-3^) to ensure both the structural stability and increased sensitivity. While IgA^gu1-3^ performed the best in terms of binding kinetics based on SPR results, it could not be used for the FP measurements, because only minor alterations in the fluorescence anisotropy upon target binding was detectable. Therefore, the shorter form, IgA^gu1-3-3^, was used, which still had the proper dissociation constant (10.4 nM) for SIgA determination in saliva, where its typical concentration is 0.6–1.2 µM. The FP measurement showed good correlation with the antibody-based ELISA assay, indicating the potential application of the aptamer-based approach for SIgA detection.

## 4. Aptamers for IgM Detection

The recognition of the alteration on cell surfaces might be a key step for cancer detection since gene mutations—originated from cancers—cause aberration in cell membrane receptor expression and function. With efficient molecular probes, early diagnostic of cancers might be established, based on the study of cell membrane receptors.

**Figure 3:**
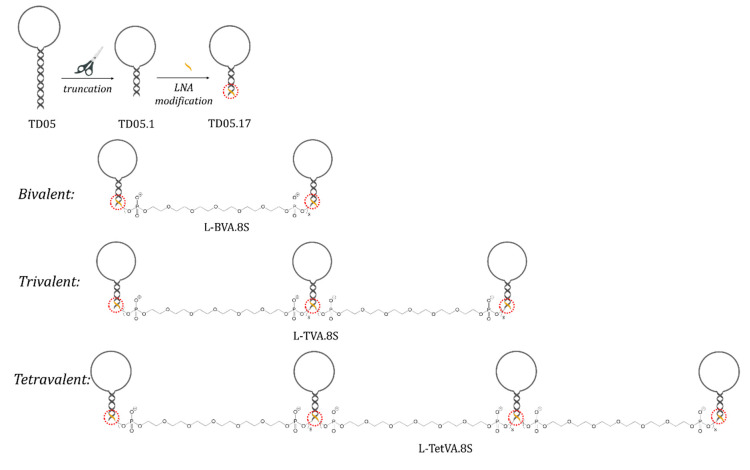
Evolution of TD05 aptamer. The previously reported TD05 aptamer was shortened with 10 nucleotides by truncation (TD05.1) and three bases have been changed to locked nucleic acid base pairs (TD05.17) (marked yellow in dashed, red circle). TD05.17 aptamers were cross-linked with PEG linkers to form bivalent (L-BVA.8S), trivalent (L-TVA.8S) and tetravalent (L-TetVA.8S) aptamers to improve the nucleic acid stability, selectivity toward mIgM and the dissociation constant [25,55].

Tang and his coworkers selected the first immunoglobulin M specific aptamer for the detection of Ramos cells, a B-cell lymphoma cell line, without identifying the particular target of the aptamer on the surface of the cell [25]. The selected TD05 aptamer (Figure 3) showed high specificity towards Ramos cells and no cross-reactivity was observed with other type of lymphoma cells, such as CCRF-CEM (acute lymphoblastic leukemia cell line), Toledo cells or other type of bone marrow cells. To identify the membrane protein target of the aptamer, a photochemically-modified nucleic acid strand was covalently cross-linked to its target on the surface of the cell. The protein–aptamer complex was enriched by magnetic bead-based extraction and the collected protein was analyzed by mass spectrometry, identified by database search and reconfirmed by the purified antibody and the selected aptamer interaction. This revealed that the TD05 aptamer binds selectively to the membrane bound heavy chain of the immunoglobulin M antibody (IGHM), which is a biomarker for the development of Burkitt’s lymphoma [54]. However, the TD05 aptamer was not able to discriminate between membrane bound and circulating forms of IgM and could not be used under physiological conditions, because the aptamer lost its affinity and stability at physiological temperature in human serum. To overcome this drawback, several modifications have been made to increase specificity and resistance in physiological conditions. Firstly, the stem length of the hairpin structured aptamer was decreased with ten nucleotides to result a more stable secondary structure (TD05.1), which fits better to the binding site of the protein. In addition, carefully chosen LNA bases have been incorporated at the 3′ end (TD05.17) to increase the melting temperature, nuclease stability and the stability of the folded form of the aptamer. With these modifications the K_D_ value was decreased 10 folds at 4 °C, but remained >10,000 nM at 37 °C. Accordingly, for in vivo application, the dissociation had to be improved further, which was achieved with multivalent analogs synthetized with poly-ethylene-glycol (PEG) linkers to crosslink membrane IgMs (mIgM) in the B-cell receptors at the cell surface. Divalent (L-BVA.8S), trivalent (L-TVA.8S) and tetravalent (L-TetVA.8S) aptamer scaffolds have been synthetized to decrease the K_D_ to 6222, 256 and 272 nM, respectively, at 37 °C. In addition, the half-life of the tetravalent scaffold in human serum was increased to 8.75 h and no interaction with soluble IgM was detected [55].

Using TD05 aptamers, Zheng et al. measured the reorganization of the IgM and IgD antibodies on the surface of the Ramos cells with branched proximity hybridization assay (bPHA), which ensures linear amplification upon proximity of the target molecules. The TD05 aptamer was modified either on the 5′ or the 3′ end, resulting TD05- and TD05+ nucleic acid strands, without changing the binding affinity of the aptamer. The Ramos cells were incubated with the elongated TD05+ and TD05- aptamer pair, and, upon hybridization, a pair of Z-DNA was added serving as a bridge for pre-amplifier, which was followed by the hybridization of amplifier and fluorescent label probe for signal amplification based on the proximity of mIgM within the nanometer resolution. With this method, the re-organization of the antibodies on Ramos cell was shown: IgD and IgM were segregated apart on resting B-cell, but after stimulation of the B-cell concatenation was observed [56].

The TD05.1 aptamer was chosen by Liu et al. for spatially and quantitatively detect membrane bound IgM molecules on Ramos cells with silver nanoparticles [57]. The 5′ end of the aptamer was modified by eight cytosine, because cytosine is favored to anchor silver clusters. By this, the mIgM distribution can be localized with confocal fluorescence microscopy and the amount of mIgM could be quantified with ICP-MS.

TD05.1 aptamer was also used to develop a chemical strategy for the glycosylation mapping of mIgM antibodies on live cells. For this purpose, the aptamer was modified at its 3′ end with cyclooctyne, which did not influence the selectivity of the aptamer for mIgMs at the surface of Ramos cells. The cells were cultured with N-azidoacetylmannosamine to generate an N-azidoacetylneuraminic acid at the cell surface during glycosylation that, after azide modification, could react with the cyclooctyne modification of the aptamer. With this strategy, the glycans could be localized within 1–3 nm of the 3′ terminus of the IgM-bound aptamer [58].

TD05.1 aptamer was used in distinct cell type differentiation in a highly multiplexed manner, based on the mIgM bound aptamer on single cell surface and simultaneous transcriptome profiling [59]. An aptamer pool and heterogeneous cell samples were incubated, where all the aptamers were modified with a poly-A tail on the 3′ end using polyadenylation. The aptamer decorated cells were co-encapsulated with barcoded beads, after lysis the aptamers and mRNAs were able to hybridize to the beads through the poly-A tail. Upon RT-PCR, the droplets contained the cell specific barcoded aptamers and mRNAs, which allowed the cells to be pooled, sequenced and evaluate epitope profiles according to the barcodes. Therefore, this method could be used to discriminate Ramos cells from other kind of cells based on selective aptamer binding and differences in gene expression.

Zumrut et al. introduced a ligand-guided selection (LIGS) strategy for identifying specific aptamers for a predetermined epitope of the target protein. The model was built up to select aptamers for the IgM expressed on Burkitt’s lymphoma cell, the same target as of TD05. The SELEX process was followed by LIGS, where aptamer candidates were pre-incubated with the target cells, followed by removal of unbound aptamers. Then, the anti-IgM antibodies were competed for the pre-determined epitopes of mIgM and eluted the aptamers, which shared the same binding site [26]. However, the affinity of these aptamers was not yet appropriate for diagnostic applications and therefore structural optimization was performed. R1 aptamer (Table 3) was chosen for further development, because it had high ability to recognize IgM on the cell surface and block the antigen binding region of anti-IgM antibodies. The R1 aptamer was truncated at the 3′ (R1.1) and 5′ (R1.2) ends to remove primer regions, which has a limited role in binding to the target protein. The shorter form (R1.2) could not be displaced by anti-IgM, due to its short length and conformational stability, therefore the aptamer could be used for the detection of sIgM and mIgM in ex vivo experiments. The lack of proper differentiation between sIgM and mIgM is based on the secondary ligand selection during LIGS, since anti-IgM antibody was also able to identically bind to the epitope of sIgM and mIgM, a feature which was descended to the aptamer [60,61]. Dimeric aptamers have also been synthetized with PEG-linkers in different length (3, 5 or 7 spacer molecules); the dimeric R1.2 aptamer showed specific binding to the mIgM positive cells, therefore it can be used as a diagnostic tool and therapeutic agent in samples of Waldenström’s macroglobulinemia patents [62]. Comprehensive structural analysis of R1.2 have been carried out, examining the role of the G-quadruplex (GQ) structures. The GQ structure with affinity constant to the target in the nanomolar range has been identified in tris(hydroxyamino)-methane solutions with elevated K^+^ level. This is in agreement with previously published papers that the GQ assembly is induced most effectively in K^+^-rich environment (>50 mM) [63,64,65].

## 5. Detection of IgGs with Aptamers

Aptamers against IgG molecules are in the center of attention owing to their broad range of applicability in bioanalytical assays. The selected aptamers can be classified in two main groups: Fc region specific aptamers and Fab specific aptamers. The Fc binding aptamers are most often used as secondary labeling agent (tracer) in affinity assays or for affinity purification of antibodies, while Fab binding aptamers in specific target determinations.

### 5.1. Fc Domain Specific Aptamers

The aim of the Fc specific aptamers is to recognize epitopes which are commonly shared in the IgG domain of a certain species. These antibody binding aptamers are applicable for affinity chromatography [66], small protein detection [67], visualization of cells and organisms [68] and signal amplifiers [69,70,71], which would cause enhanced sensitivity and improved limit of detection and limit of quantitation.

**Figure 4:**
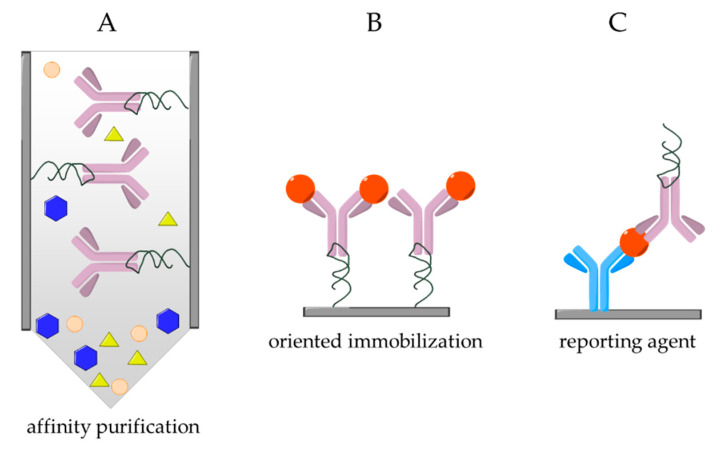
Possible applications of IgG specific aptamers: affinity purification (**A)** [66,72], oriented immobilization (**B**) [67] and as reporting agent (**C**) [69,71] (e.g., fluorescence detection and PCR amplification).

A 2′-fluoropyrimidine-modified aptamer for human IgG1 have been selected and analyzed by several research groups. The selected Apt8-2 aptamer showed high specificity to the Fc domain of human IgG (hFc1), but no cross reactivity to IgGs from other species was detected (Table 4) [17]. X-ray crystallography of the aptamer–hFc1 complex elucidated that the RNA aptamer is bound to the less-positively charged surface of the Fc region of the hIgG in contrast with the assumption that the RNA binding area takes place on the positively charged surface of the protein via electrostatic interaction. It was shown that the formation of the aptamer–antibody complex is supported by multiple weak secondary interactions as van der Waals interactions and hydrogen bonds. In addition, according to the investigated crystal structure, no aptamer induced structural changes were detected on the hIgG1 [72,73]. *Ab initio* FMO calculation have also been performed to better understand the high specificity and affinity of the Apt8-2 aptamer. It was shown that the G7 (stands for guanine, the 7th base from the 5’ end of the aptamer) nucleotide performed the strongest interaction energy with the Lys340 on the Fc region of the IgG. This energy is partially based on the electrostatic interaction of the negatively charged RNA and the positively charged protein. The interaction between the G7 nucleotide and the Lys340 could be the driving force of the RNA aptamer binding. However, the high degree of the shape complementarity suggests the presence of the hydrogen bonds, π–π stacking and CH–π interactions, which might be responsible for the high selectivity of the RNA aptamer [74]. The X-ray crystal structure revealed that the oxygen atoms of the phosphate backbone is bound to divalent ions, such as Ca^2+^ and Mg^2+^, in the major groove to stabilize the folded structure of the RNA aptamer. Therefore, the specific three-dimensional structure of the aptamer can be reversibly destabilized with chelate complexing agent [72]. Since the process is fully reversible and applied under neutral conditions, Apt8-2 seems to be an ideal recognition element in affinity chromatography (Figure 4A), i.e., as the elution step is very mild, unlike in case of using Protein A/G, the active conformation of the therapeutic antibodies can be maintained after purification/separation. Moreover, the Apt8-2 aptamer was further manipulated by ribose 2′-modification to generate Apt131, which was resistant in alkaline conditions (up to pH = 14), making the aptamer-modified resin reusable numerous times with equivalent binding capacity as Protein A [66]. The aptamers could also be used for oriented IgG immobilization in chemical sensors. A relevant example is the confinement of thiol-labeled RNA aptamers on the surface of gold coated silica nanoparticles to immobilize hIgG1 molecule exposing their binding domains for fibrinogen binding and subsequent optical detection (Figure 4B) [67]. The same Apt8-2 had also been used in a novel method for aptamer-directed conjugation of DNA to IgG antibodies [68]. Two methods of nucleic acid conjugation to antibody have been investigated: (i) templated conjugation method, when a second DNA strand is guided to react with the antibody by the aptamer; and (ii) direct method, when the aptamer itself is reacted with the target antibody. Especially, the direct conjugation of the aptamer has possible uses in immune-PCR or ELISA for in vitro detection.

Apparently, the highest affinity aptamer selected for Fc domain is an RNA aptamer, called R18, which was found to have an equilibrium dissociation constant of 15 pM [6]. This targets rabbit IgG; therefore, it can be used as a secondary antibody-like aptamer against rIgG. According to the analysis and from the predicted structures, it was shown that one of the loops plays an important role in the binding activities, especially a single adenosine base in the 49th position, because two orders of magnitude increase was resulted when this adenosine was changed to guanosine. Truncation experiments were carried out showing that nearly the entire R18 aptamer is responsible for the binding toward the rIgG. The aptamer was capable to distinguish between the native structure of IgG and the denatured structure caused by SDS. The R18 aptamer showed a great performance in affinity assays with clear benefits over conventional ELISA systems by enabling the use of PCR for the detection. Thus, in the method coining the name of immuno-aptamer PCR (iaPCR), the usual ELISA protocol is followed, but the R18 aptamer replacing a secondary antibody enables in the last step the use of qRT-PCR for amplified detection (Figure 4C) [69]. The process differs from the immuno-PCR, because in iaPCR the covalent modification of the tracer antibody is circumvented, which simplifies and improves the sensitivity of the assay. As low as 16 attomoles could be detected in a relevant assay targeting VEGF (vascular endothelial growth factor), which is ca. 100 time lower than with commercially available antibody-based ELISAs. It is important to note that the aptamer has shown no cross-reactivity for mouse, rat, human, chicken, sheep and rat IgGs. The iaPCR has also been used for the detection tumor necrosis factor-α (TNF-α) [70] where the conventional ELISAs are likely to fail due to the low concentration of TNF-α (average concentration of 100 fM). However, using iaPCR and changing the solid support from plate to magnetic beads, a limit of detection of 10 fM was achieved. The main limitation of this methodology is the relatively long time (30–60 min) required for the PCR detection [70].

Besides the PCR amplification, the inherent properties of nucleic acid strands could be used in charge modulation-based assays, i.e., the negative charge of the aptamers could be exploited for the indirect detection of the binding of various target. For instance, the change in surface charge upon the binding of R18 aptamer could be detected by silicon nanowire field-effect transistor (SiNW-FET). This enabled the label-free determination of 6X-histidine and amyloid *β* 1-42 (A*β* 1-42) in direct and sandwich assay formats, respectively, through aptamer labeling of rIgG (Figure 4C) [71]. The negatively charged R18 aptamer amplified and stabilized the signal of the immunoassays lowering the LOD and LOQ as the conductance of the nanowires was significantly reduced by the increase in the negative surface charge. The developed detection methodology promises broad applicability for different targets where the final aptamer labeling induces significant surface charge density change, e.g., Alzheimer’ disease markers.

The nonproteinaceous aptamers enable in a particular, but very relevant example the detection of protein targets by using non-selective protein dyes, i.e., R18 aptamer could be used for sensitive detection of rIgG employing Coomassie Brilliant Blue (CBB) dying followed by optical detection [75]. Apparently, such dyes able to interact with the protein targets, but not with the capture aptamers, and can be used as a cost-effective alternative of labeled tracer antibodies.

Liao and his coworkers selected aptamers against mouse IgG1, mouse IgG2 and mouse anti-HBV IgG with excellent selectivity, without interaction toward albumin, egg white protein and fat free powdered milk in dot-blot assay. The aptamer was coupled with a dsDNA served as a template for asymmetric polymerase chain reaction (A-PCR) [11]. In contrast with the method of Yoshida et al. [69,70], hybridization between the template and the primer strand was not required, making it more universal and effective. According to the results, the attachment of the dsDNA retained the specific binding of the aptamer to the antibodies and supports the amplification by PCR to detect aptamer binding. As 10^−3^–10^−6^ ng of IgG could be determined with high sensitivity, and the method is adaptable to detect proteins, peptides, nucleic acids, toxins and ions with the attachment of the DNA template to the aptamer and making quantitative analysis with PCR [11].

The previously mentioned aptamers showed great selectivity, although some methods require universal aptamers, which bind common regions of antibodies in the Fc domain. A novel SELEX method based on target replacement strategy offers further versatility to the selection, i.e., the IgG subclasses (anti-Hbs IgG, mouse IgG Fc, IgG1 and IgG2a) were sequentially replaced in every two SELEX cycle to select aptamers, which were able to bind in the common sites of the IgG subclasses, but were highly discriminative against trypsin, solcoseryl albumin and human serum. These universal aptamers might serve as more general biomedical probes in analytical applications, such as immune reaction-based analysis and antibody purification with binding characteristics similar to protein A/G [5].

### 5.2. Fab Domain Specific Aptamers

Since the Fc domains are shared in the immunoglobulin G antibodies of a certain species, the selection of aptamers for the Fab region must exclude Fc binding aptamers. Several approaches have been proposed to this end, including counterselection for the Fc domain and masking the Fc domain with antibody binding proteins, as Protein A or Protein G. The selected aptamers were used in quantitative detection of disease specific antibodies (Table 5).

Aptamers (GQCL-4.7 and SSL_30_-2.5) against antigen binding domain of anti-ferritin antibody were selected [76], where a counter-selection was obtained in every cycle with an unrelated antibody from the same species to eliminate the aptamer candidates specific for the Fc domain. The binding location was confirmed with a competitive assay, i.e., the aptamers competed with ferritin for the anti-ferritin antibody binding site.

The selection of Fc binding aptamers was avoided by masking the Fc fragment of the antibody of interest with Protein G. A modified SELEX method was implemented by Muharemagic et al., where the target antibody was immobilized on Protein G-modified magnetic beads and the immobilization has concealed the Fc domain from binding aptamers [19]. Neutralizing antibodies (nAb) were chosen as the target of the selections, which are generated upon repetitive administration of oncolytic viruses, resulting in a diminished anticancer effect of the virus. Upon binding of the aptamer candidates to the Fab region of the neutralizing antibodies, they inhibited the interaction between the vesicular stomatitis virus (VSV) and nAbs, thereby the anticancer effect of the virus was preserved. This allowed efficient cancer cell infection in the presence of nAbs and consequently enhanced in vivo survival. The shielding effect of the aptamers was confirmed by a bifunctional electrochemical (cyclic voltammetry and electrochemical impedance spectroscopy) immunosensor, making possible the quantitative detection of VSV with high sensitivity, selectivity, low cost and short analysis time. With multivalent aptamers (dimeric and tetrameric), the virus aggregation was further decreased, increasing the infectivity and stability in human serum [77].

**Table 5:** Fab domain specific aptamers for different antibody targets with their sequences and K_D_ values.

Target	Name	Sequence (5′-3′)	K_D_ (nM)	Ref.
**anti-ferritin antibody H107**	GQCL-4.7	GGG ACC GUC UCG AGG GAC GGG AGG GGG AUG GAA GCG AGA CGG UCC C	1600	[76]
	SSL_30_	GGG AUG CUU CGG CAU CCC GGC CUA GGU GAG ACU GGA AGA CAU GAU GGU ACC GCU UCG GCG GUA CGU AAG CUU	64	
**VSV neutralizing antibody**	C5S	TGT GCC AAA GAG AGT GGT GGG GGG GTG GGC GGA ACT CGC G	NA	[19]
	C9S	ACC GCC TTC CAC CGT TCT CCA CCA CCC CTC AAA CAA CCC T	NA	
**anti-D allo-antibody**	No. 2	AGA GAC GGA CAC AGG ATG AGC GGT GCA GGG GGG GCG GAG AAG AGG TTG AGG GGA GCG GGT CCT TCC CCA AGA CAG CAT	51.46	[18]
	No. 7	AGA GAC GGA CAC AGG ATG AGC GGC GCA GGG GGG GCG GAG AAG AGG TTG AGG GGA GCG GGT CCT TCC CCA AGA CAG CAT CCA	543.3	
	No. 8	AGA GAC GGA CAC AGG ATG AGC GCA GGG GGG GCG GAG AAG AGG TTG AGG GGA GCG GGT CCT TCC CCA AGA CAG CAT CCA	403.7	
**bevacizumab**	-	GCG GTT GGT GGT AGT TAC GTT CGC	130	[78]
**rituximab**	RA2	TGG GGG TAG GAT TGT GGT TGG CTT TAA TTG CTT TGG TGG T	394	[20]
	RA3	GGG GGT GAG GAT TGT GGT TTG GCT TAT TGG TTT GCT GGT G	713	
	RA4	TAT ACT GGG CCG TGC GTG ACT TTT CCG TGC TGC ATG AGA G	354	
**C595**	25 base long	GAA CTG AGG TGC TTT CCC AAA ACC T	50	[12]
	72 base long	GGG AGA CAA GAA TAA ACG CTC AAG AAC TGA GGT GCT TTC CCA AAA CCT TTC GAC AGG AGG CTC ACA ACA GGC	667	

Rhesus (Rh) is one of the most common blood group systems, where two main groups are distinguished: RhD-positive and RhD-negative. When RhD-negative persons are exposed to RhD-positive blood, they produce anti-D IgG antibodies, causing delayed hemolytic transfusion reactions. As a treatment, aptamers can be applied for blocking the anti-D allo-antibodies and RhD antigens combination. Zhang and his co-workers selected aptamer candidates, for the Fab domain of the anti-D allo-antibodies, while blocking the Fc domain with Protein A. To detect the efficiency of the aptamers hemagglutination inhibition assay was performed, when anti-D antibodies were completely neutralized even by 50 pmol of aptamer (Nos. 2 and 7 in Table 5) [18]. In contrast, when ssDNA aptamers were selected to the RhD antigen, the optimal concentration for the anti-RhD-mediated RBC agglutination was 500 pmol [79].

Aptamers are promising tools also for the quantification and qualification of antibody-based biopharmaticals. Highly selective, sensitive, accurate and high-throughput analysis was introduced for bevacizumab detection, which is a humanized monoclonal antibody against vascular growth factor A to treat several cancer types. However, the concentration of bevacizumab has to be carefully determined to avoid side effects while preserving activity. For this purpose, a novel method was introduced by Yamada et al. Anti-idiotype DNA aptamer was selected for the complementary determining region (CDR) of the bevacizumab and was covalently immobilized to the microtiter plate. Bevacizumab from plasma samples interacted with the aptamer and a HRP conjugated Protein A was used as tracer for the colorimetric detection (ELAA—enzyme-linked aptamer assay). The dynamic range of the method is 5–500 ng/mL (34–3356 pM), which is comparable with the FDA approved ELISA process [78]. The same aptamer was used in affinity purification–high temperature reversed phase liquid chromatography (HP-RTLC) with fluorescence detection for bevacizumab determination from plasma samples from patients with lung cancer. A sharp, single peak was detected within 10 min, when bevacizumab was pre-extracted with anti-idiotype DNA aptamer-modified magnetic beads [80]. However, the limit of detection was 0.15 µg/mL (1 nM), which is ca. 2 orders of magnitude higher than the ELAA method (2.06 ng/mL (13.8 nM)). Another biopharmaceutically relevant antibody in cancer treatment, rituximab—a chimeric monoclonal anti-CD20 antibody—was also the target of aptamer selections. Six aptamer candidates (RA1–6), specific to the Fab domain of the antibody were selected, with dissociation constant of 354-887 nM. Despite the lower affinity, the aptamers were able to detect conformational changes of rituximab upon UV exposure, mechanical stress and storage at different temperatures. Thus, RA2, RA3 and RA4 aptamers can be used as an orthogonal method to detect changes in the antibody during operation and storage. Moreover, the aptamers showed high specificity in ELASA (enzyme linked apta-sorbent assay) method, without cross-reactivity toward glycosylated Fc domain of rituximab (Fc lyc), recombinantly expressed Fc/2 (Fc/2), adalimumab (adalim), bevacizumab (bevac) and etanercept (etan) [20].

In a series of cases aptamers with higher binding affinity were found for the antigen-binding sites of antibodies than that of the natural antigen, thereby they were able to displace the antigen from the antibody binding sites in competitive ELISA method. A relevant example is the aptamer selected against C595, an anti-MUCI IgG3 monoclonal antibody [12]. According to the molecular model of C595, the antigen binding pocket is negatively charged due to three aspartate residues, which seemed to be essential for the MUC-1 antigen docking based on electrostatic interactions with arginine. In contrast, the binding of the aptamer was dominated by hydrophobic interactions and hydrogen bonds through the basis of the nucleic acid strands, overcompensating the electrostatic repulsion of the negatively charged phosphate backbone of the aptamer.

Several aptamers have been selected against autoantibodies (AABs) in an attempt to downregulate the body’s immune response to its own tissues. These aptamers usually block the Fab domain of the antibody, thereby inhibit the binding between the autoantibody and the target, such as proteins, DNA or receptors. Aptamers were selected to detect Multiple Sclerosis (MS) [13,14], Systematic Lupus Erythematosus (SLE) [81], Dilated Cardiomyopathy (DCM), Chagas’ Cardiomyopathy (ChCM), Peripartum Cardiomyopathy (PPCM) [82,83,84,85,86,87,88,89], Extreme Insulin Resistance (type B) (T2DM) [90,91] and Myastenia gravis (MG) [92,93,94,95,96] with high affinity (K_D_ ≈ 1.2–75 nM) at an early stage of the diseases. Moreover, 2′-F-Py (2′-fluoro pyrimidine) and 2′-A-Py (2′-amino pyrimidine) bases were widely used to increase the nuclease resistance of the aptamer. The aptamers targeting autoantibodies and their binding properties were summarized in Table 6.

Aptamers were also used to detect toxoplasmosis, which is an infection caused by a parasite, through the antibodies (IgG and IgM) produced in response to the infection by selecting aptamers recognizing their relevant paratopes (Table 7). A quantum dot-labeled dual aptamer-based biosensor (Q-DAS) was developed by Luo and his coworkers for the detection of toxoplasma IgG antibodies in an aptamer sandwich complex. TGA6 aptamer was immobilized on the surface of a plate to capture the antitoxoplasma IgG, followed by a washing step and the introduction of the quantum dot-labeled TGA7 aptamer to form a TGA6/toxoplasma IgG/TGA7-QD complex. The measured fluorescent signal was linear within 0.5-500 IU, with an LOD of 0.1 IU. The steric crowding is a critical factor in the IgG binding even on the planar surface and on the curved surface of the quantum dots as well, therefore a five-base-pair-long oligo-T chain was added to reduce the steric hindrance. The Q-DAS biosensor showed great specificity against cytomegalovirus antibody, syphilis antibody, rubella antibody, antinuclear antibody, rheumatoid factor albumin, fibrinogen, IgE and IgM. The sensor was tested to detect clinical serum samples, and the results were compared with the Sabin–Feldman DT assay reference method, where 183 of 189 (95.9%) negative samples and 61 of 67 (91%) positive samples were accurately reported, with 6.5 IU cut-off. Therefore, the Q-DAS biosensor is a promising candidate for clinical toxoplasma detection at an early stage to reduce the spontaneous abortion, fetal death and birth defects [15].

## 6. IgE Detection with Aptamers

Immunoglobulin E plays an important role in allergic diseases and pathogenesis of other disorders. Upon infection, IgE is produced as a response to an allergen, followed by antibody bonding to the FcεR receptors on the mast cells. Cross-linking of the bound IgE is occurred by the re-exposure of the allergen, resulting the release of mediators, such as histamine, lipid mediators and cytokines [38]. Therefore, blocking the interactions with Fc receptors may be used in the treatment of allergic responses while the quantitative measurement of IgE in body fluids can be applied for diagnosis. Several aptamers have been selected for the Fc region of the immunoglobulin E, however, the most frequently used two sequences were published by Wiegand et al., as they were shown to have excellent K_D_ values, i.e. 30 and 10 nM, respectively (Table 8). Another selection strategy that approached this affintiy (average K_D_ of 29 nM) was based on capillary electrophoresis as proposed by the Bowser group [9]. The binding of the putative aptamers to the larger size IgE could be detected through the change in the electrophoretic mobility with respect of free aptamers. The main advantage of this approach is that required only four selection rounds and by that reduced the selection time from 4–6 weeks to 2–4 days. The selection process could be further integrated by using micro free flow electrophoresis, which again ensured aptamer selection in four days with low nM average dissociation constants for IgE, without target immobilization, long incubation time and negative selection [10]. In terms of selection time reduction, the performance of microfluidics is hard to be surpassed as the strict control in terms of temperature and integrated PCR could reduce the overall duration of the process to 4 h with a similarly drastic reduction of the reagent consumption. However, the dissociation constant of the selected IgE aptamer (83.9 nM) was not as good as in selections made by classical SELEX and CE-SELEX [22]. A fully microchip integrated version of the electrophoretic selection process by combining the bead-based aptamer selection (bead-based PCR amplification) with gel electrophoresis was improved [23], resulted in aptamers reaching a K_D_ value of 18 nM in only 3 selection rounds [24].

### 6.1. Aptamer-assisted Optical Detection of IgE

#### 6.1.1. Colorimetric Methods

**Figure 5:**
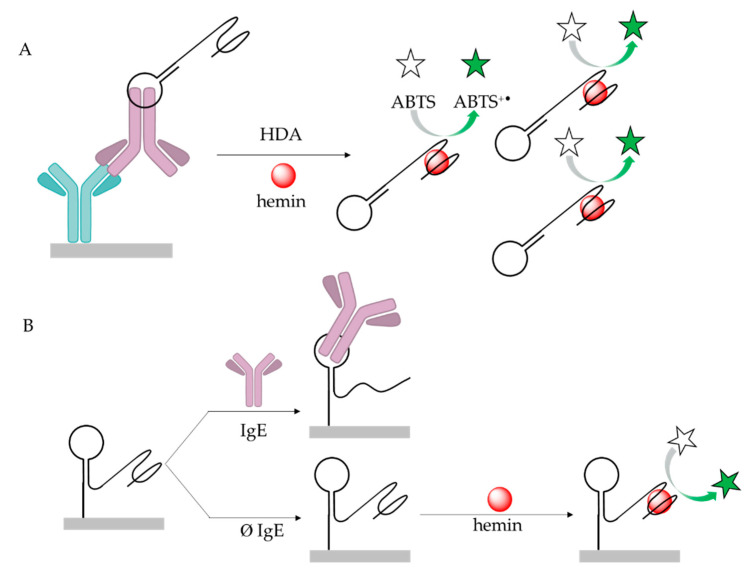
Sandwich assay based (**A**) and direct (**B**) colorimetric methods coupled with peroxidase activity of DNAzymes [98,99].

The most commonly used colorimetric methods for IgE detection are based on measuring absorbance changes induced by enzyme or nanoparticle-based labels. Perhaps the simplest opportunity is provided by the dot-blot assays using horseradish peroxidase as labels. In such assays, IgE is generally spotted on nitrocellulose membrane and a biotinylated aptamer is used for selective IgE recognition coupled with an HRP conjugate that catalysis the formation of a colored compound. Relevant examples indicate that such assays can be accomplished in ca. 40 min with LODs in the lower nanomolar range [97]. However, the conventional HRP label can be replaced by DNAzymes exhibiting peroxidase activity that can be conveniently linked to the aptamer sequence, resulting in a bifunctional probe, e.g., IgE aptamer and peroxidase-active hemin aptamer. This was implemented in a sandwich assay by using a surface immobilized anti-IgE antibody and the bifunctional aptamer probe. To enhance sensitivity asymmetric, isothermal amplification (HDA—Helicase Dependent Amplification) was used (Figure 5A). Upon amplification, hemin could bind to the hemin specific part of the bifunctional aptamer that enabled the oxidation of the ABTS (2,2′-azino-bis (3-ethylbenzothiazoline-6-sulfonic acid)) into green color, which could be monitored by a simple spectrophotometer down to 1 pM or even by naked eye down to 10 pM [98]. A simpler but similar method based on DNAzymes was also developed for solid phase enzyme-linked immunosorbent detection of IgE [99]. The aptamer and G-quadruplex structured DNAzyme were immobilized on the surface of the wells. In the absence of IgE, a hairpin structure involving the anti-IgE aptamer and a G-quadruplex structure from DNAzyme were formed, therefore hemin was able to bind to the G-quadruplex structure to catalyze the H_2_O_2_ mediated oxidation of ABTS. However, when IgE was present, the formation of the G-quadruplex is inhibited, and the absorbance reduced in an IgE concentration dependent manner (Figure 5B). This approach was shown to be applicable in urine samples with LOD of 10.5 pM, without the need of isothermal amplification or capture antibody.

A label-free homogeneous detection was introduced by Yoshida et al., with aptameric enzyme subunit (AES), where the artificial enzyme subunit was constructed from two unmodified aptamers (thrombin binding aptamer and IgE binding aptamer). In the absence of IgE, the two aptamers were folded in a G-quartet structure, which by binding thrombin inhibited the clot formation of fibrinogen. However, in the present of IgE, the G-quadruplex structure was destroyed resulting clot formation, and the clotting time could be measured with an automated fibrometer [100,101].

In colorimetric detections, nanoparticles are also frequently used as reporting agents including silver [102], gold [103,104] and carbon nanoparticles [105] and aptamer-labeled bacteriophages [106]. Aptamer-modified silver nanoparticles were used for rapid, visual IgE detection, where anti-human IgE antibodies were spotted on solid support to capture IgE and form antibody/IgE/aptamer–AgNP complex. The signal was further enhanced by reductive silver deposition creating a pattern of dark spots, which were localized and quantified by an array scanner. The method was able to detect IgE down to ca. 20 ng/mL (105 pM) in a cost effective, easy-to-use manner [102].

**Figure 6:**
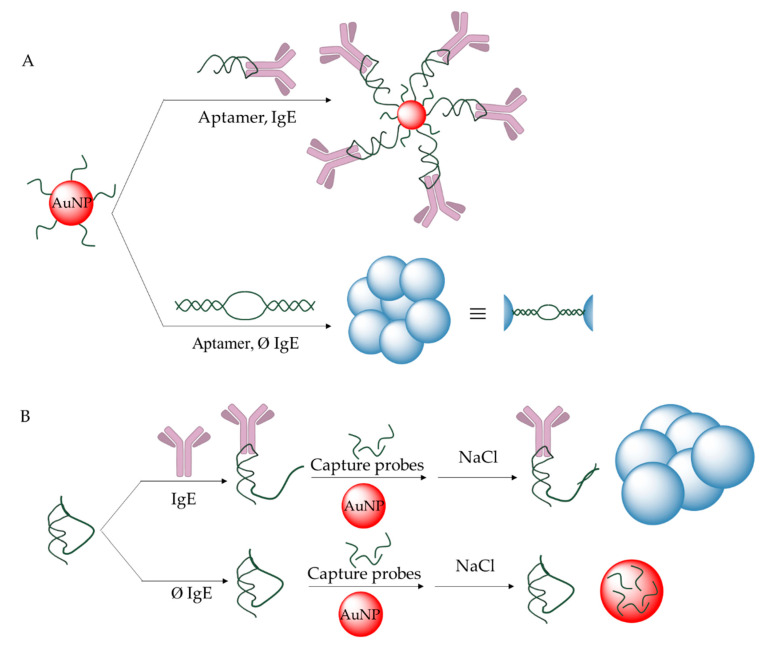
Gold nanoparticle-based colorimetric detection of IgE in solution phase [103,104]. The aggregation of gold nanoparticles could be induced by the absence (**A**) or the presence (**B**) of IgE.

An interesting AuNP aggregation mechanism introduced by Wu et al. [103] is based on marked conformational changes induced by the IgE binding to the AuNP confined aptamer strands. Generally, the DNA-modified AuNPs are very stable in solution due to the electrostatic repulsion of their negatively charged surfaces and this stabilizing effect persists even at high ionic strengths. In case of IgE aptamers, however, a salt induced aggregation can readily occur due to intermolecular hybridization of the AuNP modifying aptamer strands that was called hairpin aptamer sticky-end pairing effect by the authors. Upon IgE binding, the change in the aptamer conformation prevents/disrupts this interparticle hybridization event and as such the aggregation of the nanoparticles even at high concentrations. Since the aggregated and non-aggregated forms of the aptamer-modified AuNPs have markedly different colors, the method provides sensitive means for IgE detection down to ca. 20 pM LOD with wide linear range (Figure 6A). An innovative alternative to this method used a short single-stranded DNA non-covalently attached to the surface of AuNPs to protect them from aggregation, i.e., in the absence of IgE the capture probes kept the stability of the particles [104]. An elongated aptamer probe was designed such that the elongation was able to hybridize to the loop structure of the aptamer, creating a pseudoknot aptamer. In the presence of IgE, the pseudoknot strand dehybridized from the loop region of the aptamer and hybridized to the capture probe, leaving the surface of the AuNP unprotected, as indicated by the aggregation of the gold nanoparticles (Figure 6B). The shift of the absorbance spectra from red to blue depending of IgE concentration resulted in a 0.2 nM LOD.

A lateral flow sandwich assay was developed for colorimetric determination of IgE using a chemically-modified bacteriophage [106]. In vivo biotinylated peptide (AviTag) was fused to the tail pIII protein of M13 phage to link the biotinylated IgE aptamer through neutravidin. Additionally, the pVIII core protein was covalently modified with HRP to catalyze the oxidation of TMB (3′,3,5,5′-tetramethylbenzidine) for colorimetric detection. The lateral flow assay contained anti-IgE at the test line, which ensured the selective recognition of IgE, whereas at the control line anti-M13 antibodies were immobilized. With the modified bacteriophage a limit of detection, 0.68 pM was reached.

#### 6.1.2. Surface Plasmon Resonance Based Detection

Most of the surface plasmon resonance measurements aimed at the determination of the binding kinetics, affinity and selectivity of the IgE aptamers. One of the best studied IgE aptamers in this respect is the D17.4 IgE aptamer (Table 8), introduced by Wiegand et al. [7]. The dissociation equilibrium constant for the full length IgE protein shows at first sight a very high variation with values ranging between 320 pM and 270 nM (Table 9). This variation reveals in fact how sensitive is the aptamer-target interaction to the immobilization conditions, probe design and experimental conditions, i.e.., the immobilized terminus of the aptamer, the length of the linker between the surface of the supporter and the stem-loop structure, the surface chemistry and the surface density of the immobilized aptamer are all factors that need to be considered. It seems that on average the dissociation equilibrium coefficient of the IgE–aptamer complex is higher if the aptamer is immobilized to the surface through the 5′ end than the 3′ end. The published immobilization methods for this aptamer are very diverse but conclude on the importance of a mixed layer for appropriately large spatial separation of the aptamers. This was achieved for instance by co-immobilization of carboxyl capped and uncapped polyethyleneglycol chains followed by EDC/NHS activation of the amino-labeled aptamers [107,108] or by using streptavidin–biotin interaction with biotin-labeled aptamers [109,110]. In addition, direct immobilization of the thiol-modified aptamers on the gold surface in a mixed monolayer with cysteamine or thiol-modified alcohols can also be used, in one- or multi-step reactions [111,112,113,114,115]. The effect of the solution composition that should generally approach the selection conditions and particularly the Mg^2+^ concentration has also enormous influence on the equilibrium dissociation constant. Wiegand and his co-workers analyzed the D17.4 aptamer–full length IgE interaction in modified PBS buffer (PBS adjusted with 1 mM MgCl_2_) [7], however in subsequent studies various other buffers were used, e.g., tris(hydroxyamino)-methane [107,110], HEPES [109,116] and PBS [111,113,114] without Mg^2+^ ions. As indicated in Table 9, when Mg^2+^ have been added to the working buffer [108,112,115], the K_D_ value was under 5 nM, while, in the absence of Mg^2+^, it was a few tens of nM. To understand the kinetics also in terms of aptamer surface density a rapid and convenient assessment method based on a single injection of hexamine-ruthenium (III) solution [115] was developed. The ruthenium complex in appropriate conditions associates to the aptamer strands by electrostatic interaction fully compensating their negative charge. Thus, the surface density of aptamers can be calculated from the amount of the bound cationic complex and stoichiometry of the binding reaction. By this, the optimal conditions of the heterogeneous aptamer–IgE interaction could be revealed and accurately quantified to avoid molecular crowding within the sensing layer but preserve appropriate sensitivity.

**Figure 7:**
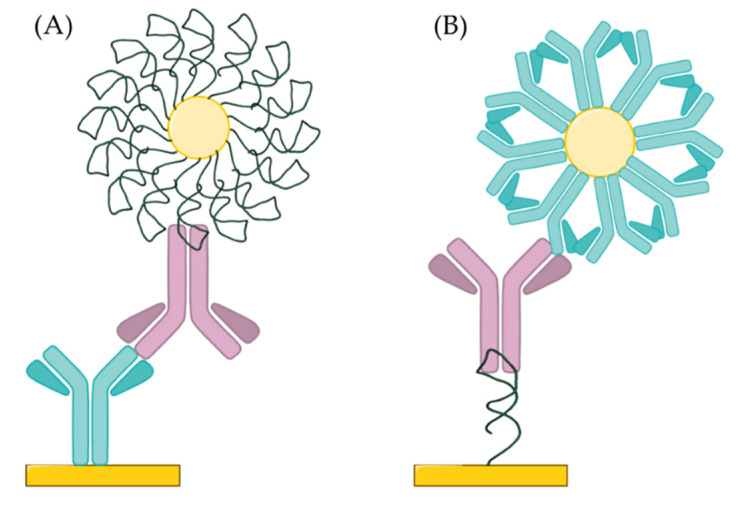
Methods for signal enhancement with aptamer (**A**) or anti-IgE (**B**) modified gold nanoparticles in SPR for IgE detection [113,117].

To enhance the sensitivity of the SPR measurements, nanoparticle-based (e.g., gold) amplification methods have been most often applied with two main variation of the sandwich assay: (i) using immobilized anti-IgE capture antibody and following the IgE binding by addition of the tracer aptamer-modified gold nanoparticles (Figure 7A); and (ii) using an immobilized capture aptamer and revealing the binding of IgE by anti-IgE-modified nanoparticles (Figure 7B). Using gold nanospheres according to method (i) improved the limit of detection of the amplification-free SPR detection to 0.001 µg/mL (5.3 pM) [117]. However, Kim et al. compared the two different methods and found, that using the aptamer/IgE/anti-IgE succesion (i) resulted in 10-fold improvement in the LOD (1 fM) compared to the second methodology [113]. The limit of detection was further improved by replacing the anti-IgE-modified gold nanospheres with gold nanorods (1 aM) due to the plasmonic coupling between the nanorod and the gold film on the substrate [118].

#### 6.1.3. Fluorescent/Luminescent Methods

**Figure 8:**
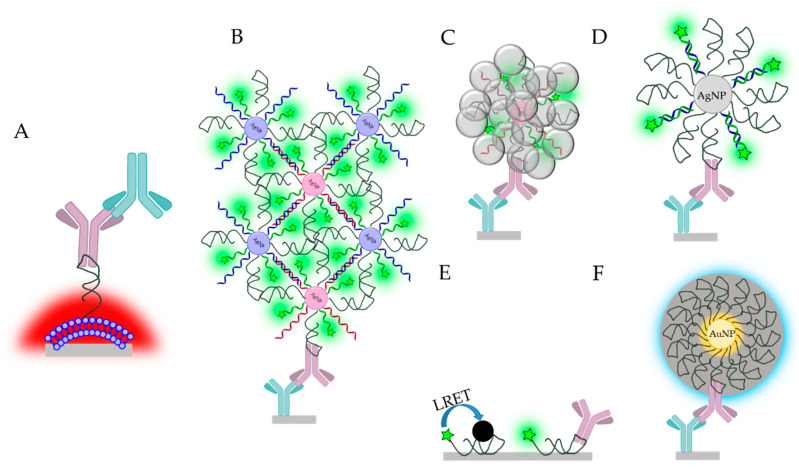
Heterogeneous fluorescence and luminescence detection-based assays [121,122,123,124,125,126] using colorimetric transition of liposomes (**A**), metal-enhanced fluorescent detection with hybrid probes (**B**), silver nanostructures (**C**) and fluorescent probes (**D**), luminescent resonance energy transfer between upconverting nanoparticles and carbon nanoparticles (**E**) and non-stripping gold nanoparticles (**F**).

In general, fluorescence provides excellent sensitivity and LOD along with high-throughput analysisusing microarray technology. Aptamer strands are typically immobilized onto the surface of glass slides, e.g., the glass slide is modified with avidin followed by the immobilization of the biotinylated aptamers [119] or the amino-modified aptamer are linked through glutaraldehyde to amino-silanized surface [120]. The surface immobilization procedure and the proper binding buffer selection could be determined by fluorescence [119] or using ^32^P radiolabeled aptamers [120]. A more sophisticated method for signaling on glass support was reported based on the use of poly(di-acetylene) (PDA) liposomes, which exhibit a colorimetric transition in response to a wide range of stimulations, including ligand–receptor binding on its surface. Thus, after the surface of the liposomes were modified with aptamers, the selective recognition of the IgE resulted in shear stress and consequently fluorescence signal increase that could be further amplified by addition of polyclonal anti-IgE antibodies to reach an LOD of 0.01 ng/mL (53 fM) (Figure 8A) [121]. This could be further improved by Li and his co-workers using ultrasensitive metal enhancement fluorescence (MEF)-based detection of IgE with an LOD of 211 fM (Figure 8B) [122]. To this end, two sets of silver nanoparticles (AgNPs) were modified with a mixture of three different nucleic acid strands, i.e., the aptamer, a fluorescent Cy3-labeled oligoA strand and oligos that were complementary to each other on the two sets of AgNPs. The assay was carried out on an anti-IgE-modified glass (through aldehyde coupling). After IgE binding to the immobilized antibody, the nanoparticle aggregates, formed by the hybridization of the two complementary oligos, were attached to the surface via the selective aptamer–IgE interaction. The MEF enhanced fluorescence resulted from the the emission resonance of Cy3 molecules coupled to the broader localized surface plasmon (LSP) of AgNP aggregates was detected using a fluorescence scanner. The method could be simplified to one set of nanoparticles and the signal enhancement could be achieved by catalytic silver deposition, however at the expense of a lower sensitivity (Figure 8C) [123]. In all cases, the metal enhancement fluorescence needed proper optimization of the distances between particles and fluorophores. In this respect, an exceptionally wide linear range was achieved with a rigid structure-based MEF method, when silver nanoparticles were modified with aptamer strand co-immobilized with an “indifferent” nucleic acid strand of a specified length (Figure 8D). The complementary, fluorescent-labeled strand could hybridize to the nanoparticle and a rigid duplex was provided, where the distance between the particle and the fluorophore is well controlled [124].

Cheap, disposable, paper-based aptasensor was developed using luminescence resonance energy transfer (LRET). Yb and, Er upconverting nanoparticles (UCNPs) modified with the IgE aptamer served as energy donor in the immunoassay, whereas carbon nanoparticles (CNPs) served as energy acceptors. In the absence of the IgE molecules, CNPs were held in the close proximity of the UCNP by π–π stacking interaction between the bases of the nucleic acid strands and the CNPs. This resulted in a quenching of the luminescence signal. However, in the presence of the IgE, the π–π stacking interaction was disrupted that triggered the luminescence signal from the UCNP (Figure 8E) [125]. The analytical performance parameters of the heterogeneous fluorescent assays are summarized in Table 10.

Several homogenous light switching methods were developed as compiled in Table 11. Since there is no need for the separation of bound and unbound fractions of the reporter molecule, the automatization of the detection processes is typically simpler and the inherent properties of the nucleic acid aptamers can be very effectivel y exploited. A relevant example is based on the use of a [Ru(phen)_2_(dppz)]^2+^ complex as indicator of the aptamer–IgE binding. The ruthenium complex is able to intercalate in the double stranded region of the aptamer in the absence of IgE. However, in the presence of IgE, the structure of the aptamer changes owing to the target–aptamer interaction, that triggers a luminescent signal change [128]. Of note, the generality of this method is restricted by the necessity to have a double stranded stem region in the aptamer structure that is “untied” upon target binding. This was circumvented by using an internal modification of the aptamer with 2-aminopurine (2AP) nucleotide analogs, where the fluorescence quantum yield depends on the base stacking interaction between 2AP and its neighbors. Thus, if the aptamer undergoes a conformation transition upon target binding, this can result in a fluorescent signal change. This strategy may provide quantitative and qualitative information regarding of the nature of the binding without any additional nucleic acid strands [129].

A method with wide applicability was published by He et al. where a mixture of fluorescent- and quencher-labeled nucleic acid strands were able to hybridize to IgE aptamers in the absence of the target molecule bringing the labels in close proximity, which results in a very low fluorescence backgorund signal. However, when the aptamer binds to IgE, the two probes are distanced to result in an intense fluorescence signal allowing for ca. 57 pM LOD [130]. 

A 5 pM limit of detection was achieved with allostery-triggered enzymatic recycling amplification, and the system was able to detect IgE with proper sensitivity, even in complex biological samples. The universal strategy was investigated with endonuclease-based target-triggered signal amplification with IgE aptamer. The highly complex sensing method ensured IgE determination in a wide dynamic concentration range [131]. Another enzymatic method was developed based on a multicomponent G-quadruplex and monopyrene-based system, where 95.4 fM LOD was achieved using the digestion by S1 nuclease that differentiates between double stranded and single stranded DNA strands [132].

Fluorescence anisotropy-based methods have also been developed for IgE detection and optimized in terms of the fluorescent label, length of the linker, ionic strength, concentration of divalent cations and temperature. Gokulrangan et al. found that the type of the fluorophore influences more the sensitivity of the assay through its local motion then the length its linker [133]. The proximity of the fluorescent dye is especially important for the sensitivity of fluorescent anisotropy assays. When it was in close proximity to the IgE molecule, much higher fluorescent polarization changes were observed, which is due to the restricted local rotation of the dye upon target binding. However, the position of modification has to be carefully selected, since the dye can also reduce the affinity of the aptamer towards its target [134]. The anisotropy measurements also led to more information on the aptamer–IgE binding in solution, apart from heterogeneous binding assays by SPR. The dissociation constant was found to be similarly influenced by the buffer composition. When the NaCl concentration was increased from 50 to 300 mM, a five-fold increase in the k_D_ value was detected, which might be due to differences in the loop conformation, essential for the IgE binding. Moreover, it was shown that the Fc domain is positively charged, thereby the binding of the aptamer to IgE is partly based on electrostatic interaction which is significantly influenced by the ionic strength [135]. Increasing the temperature resulted in an apparent increase of the k_D_, since the conformational flexibility and the dye motion is in strong correlation with the temperature [133,135].

A possibility to increase the signal-to-noise ratio of homogeneous fluorescence-based assays is to minimalize the background signal corresponding for non-specific interaction by using antibody induced quenching. To this end, the IgE aptamer was modified with FITC (fluorescein isothiocyanate) in proximity to the target-binding pocket of the aptamer. In the absence of IgE, FITC-antibody could bind to the dye and efficiently quench its fluorescence and prohibit the non-specific interaction of proteins. In the presence of IgE, the aptamer binds to IgE by outcompeting the FITC-antibody and inducing fluorescent emission. The method can be multiplexed using different fluorophores with non-overlapping emission peaks, providing a robust, simple and high-throughput option [136].

**Table 11:** Homogeneous fluorescence-based IgE assays.

Detection Principle	Linear Range (pM)	LOD (pM)	Ref.
Molecular Light Switch Complex	100–800	100	[128]
Fluorescence enhancement using a DNA aptamer	92–37,000	57	[130]
Amplification through allostery-triggered enzymatic recycling amplification	NA	5	[131]
Fluorescent oligonucleotide probe based on G-quadruplex scaffold for signal-on ultrasensitive protein assay	4.72–7560	0.095	[132]
Fluorescence anisotropy	1000–60,000	350	[133]
Fluorescence anisotropy assay	NA	20	[134]
Fluorescence protection assay	100–50,000	100	[136]
Aptamer-barcode-based assay based on instantaneous derivatization chemiluminescence coupled to magnetic beads	4.88–20,000	4.6	[137]
Competitive fluorescence quenching assay	350–35,000	170	[138]
Rapid fluorescence detection of immunoglobulin E using an aptamer switch based on a binding-induced pyrene excimer	NA	1600	[139]

#### 6.1.4. Electrochemiluminescence (ECL) Assays

A label-free, switching-off type electrochemiluminescence biosensor was developed for sensitive detection of IgE. Petal-like CdS nanoparticles were deposited on the chitosan coated glassy carbon electrode, followed by a secondary layer of chitosan and gold nanoparticle deposition to enhance and ensure stable ECL signal, owing to the high ECL efficiency of the semi-conductor particles, good conductivity and the large electroactive surface as well as aptamer loading capacity of the gold nanoparticles. IgE aptamer was absorbed on the surface of the AuNPs to ensure the selective detection of IgE. When the target molecule is introduced, the ECL signal was highly suppressed, due to the inhibited diffusion of the ECL co-reactant toward the sensor surface. The achieved limit of detection was 80 fM and the label-free method was able to measure IgE concentration in human serumdown to 1 pM concentration [140]. A competitive, turn-on type sensor has also been developed based on enzyme induced biocatalytic precipitation (BCP). A complementary strand of the IgE aptamer was immobilized on the electrode surface (CdSe/ZnS quantum dots (QDs) functionalized MoS_2_), while the aptamer along with HRP was attached to gold nanoparticles. In the absence of IgE, the nanoparticles by hybridization of the aptamer and the complementary strand attached to the electrode surface and the HRP catalyzed precipitation formation blocked the electrode surface and suppressed the ECL signal. However, the presence of IgE in the sample reduced the amount of surface bond gold nanoparticles by competition, i.e., the IgE-bound aptamers could not hybridize to the surface confined probe. This results in the reduction of the amount of precipitate formed at the electrode surface in an IgE concentration dependent manner. The process however overall leads to an increase in the ECL signal and this concept could be used to detect IgE in human serum at as low as 2 pM level, with an LOD of 180 fM in buffer solution [141].

### 6.2. Electrochemical Detection of IgE

**Figure 9:**
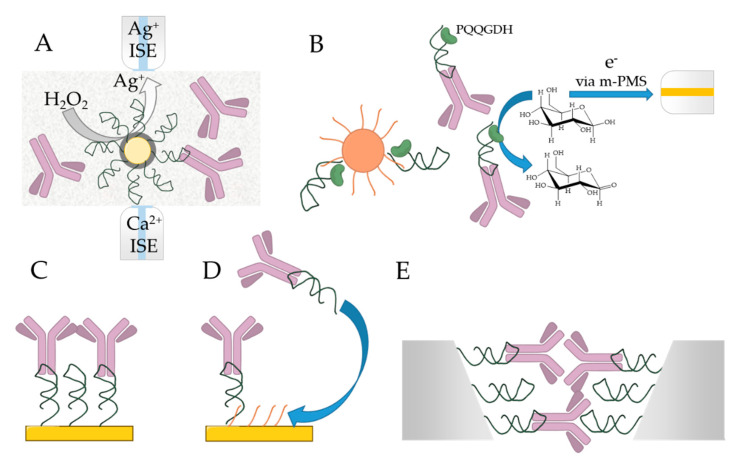
Potentiometric (**A**) [142]; amperometric (**B**) [143]; array-based impedimetric (**C**,**D**) [146,147]; and nanopore-based (**E**) [149] detection schemes of IgE based on the use of D17.4 IgE aptamer.

Electrochemical sensors by their easy miniaturization, simple and rapid detection of IgE are ideal candidates for the fabrication of PoC devices. The simplest and most cost-effective approaches are probably based on potentiometric transduction that are especially advantageous if realized on paper-based platform. In this respect, a potentiometric dot blot assay was proposed that is based on using the catalytic activity of gold nanoparticle-IgE aptamer conjugates (Figure 9A). Such nanoparticles catalyze the silver deposition on the IgE spots in a concentration dependent manner. The silver ions released by oxidative dissolution with H_2_O_2_ were measured with a silver ion-selective electrode that allowed subsequent quantitation of the IgE [142].

Amperometric measurements coupled with nanoparticle techniques have also been used to detect IgE with pyrroquinoline quinone glucose dehydrogenase (PQQGDH)-labeled aptamer strands. If an assay uses a labeled aptamer for the detection of the target molecule, the bound and unbound aptamers have to be distinguished, to eliminate false positive signals. Therefore, Fukasawa et al. developed a nanoparticle-based bound/free separation system, where complementary strands of the aptamer were immobilized on NeutrAvidin beads. In the absence of the IgE, the PQQGDH-modified aptamers could bind to its complementary strands on the beads, thereby no current change could be measured. However, in the presence of IgE, a stable aptamer–IgE complex was formed, since the stability of the aptamer–protein complex is higher than that of the aptamer–cDNA complex. While the unbound aptamer strands were trapped, the IgE–aptamer complexes were measured on the surface of a thin gold wire electrode based on the activity of the PQQGDH enzyme in the glucose containing buffer (Figure 9B) [143]. Although the LOD is relatively high (1 nM), the described method offers an appealing strategy to separate bound and unbound forms of a target.

One of the main benefits of electrochemical methods is the label-free transduction mechanisms. A relevant example is the use of polyaniline (PANI) nanowires covalently modified with aptamers for label-free, real-time, sensitive IgE detection based on relative conductance changes upon IgE binding. In the presence of IgE the conductance was increased, because the negatively charged IgE was specifically accumulated on the surface of the p-type nanowire blocked previously with BSA, a compulsory step to ensure applicability. However, the variation of the signal response was very considerable [144], which might be due to the uncertainty of the covalent modification of the nanowire with aptamer and also the complexity of the sensing mechanism. The reproducibility of the approach was improved when polypyrrole nanowires were grown to incorporate the aptamers in one step. With the described method, the batch-to-batch variation of the sensors was significantly reduced along with benefits in terms of a faster response (~20 s) and LOD (10 pM) [145].

Electrochemical impedance spectroscopy (EIS) also ensures label-free detection of IgE. Generally, the charge transfer resistance is measured, which scales with the bound protein, particularly IgE, to the aptamers confined at the electrode surface. The electrodes can be prepared conveniently by photolithography in the form of arrays, as shown by Xu et al. It was found, that in the case of EIS detection, the aptamers have genuine advantage over antibodies in terms of lowered background noise, minimal non-specific adsorption and larger relative signal (Figure 9C) [146]. Since the immobilization can influence the specific 3D structure of the aptamer, a DNA directed capture method was introduced to circumvent this problem. In the relevant proof of concept study, instead of directly immobilizing the aptamer onto the electrode surface, a complementary DNA to an elongated aptamer is immobilized, i.e., complementary to the terminal elongation of the aptamer so that the hybridization does not affect the IgE binding capability of the aptamer. For IgE measurements, the aptamer was then reacted with IgE in solution phase and the complex was captured by the complementary DNA (Figure 9D) [147]. In this detection method, steric hindrance should be largely avoided; moreover, after regeneration the same microfabricated array can be used for different target proteins, with the elongation of the respective aptamer strands coding the target protein. Contrary to the detections based on charge transfer resistance changes in the case of nanocrystalline diamond (NCD) electrodes, the binding of IgE to surface confined aptamers could be transduced via changes in the superficial capacity. With this approach, a ca. 3 orders of magnitude dynamic range and an LOD of 0.03 µg/mL (158 pM) was obtained. In addition, the sensor was highly selective for IgE even in the presence of large amount of IgG background and in human serum [148].

Another type of electrochemical label-free methodology based on chemically-modified nanopores that could reach single molecule detection capability was introduced by the group of Gu. For this purpose, glass nanopores of ca. 60 nm were modified with aptamer strands that in case of nanoconfinements have clear advantages over antibodies in terms of their small size [149]. Measuring the ion current through the nanopore in an electrolyte solution showed clear blockade events upon IgE binding. As the ca. 11-nm-diameter protein molecule bound to the aptamer-modified inner wall of the nanopore, a volume equivalent of highly conductive electrolyte from the pore was excluded, resulting in a resistance increase and consequently transport current decrease. Due to the strong binding strength of IgE–aptamer complexes, each IgE molecule binding could be detected as a stepwise decrease in the registered current (Figure 9E). The step heights however continuously decreased as the number of bound IgEs in the nanopore increased owing to the smaller relative resistance change. While single molecule detection is paradoxically not always associated with very low LODs, which is a statistical quantity [150], the method could be used for quantitative determinations based on the average time elapsed between the steps (at higher concentrations the duration between the steps is shorter) down to ca. 1 pM. However, the pre-filtration of the sample is required, since clogging can occur during measurement of complex samples.

**Figure 10:**
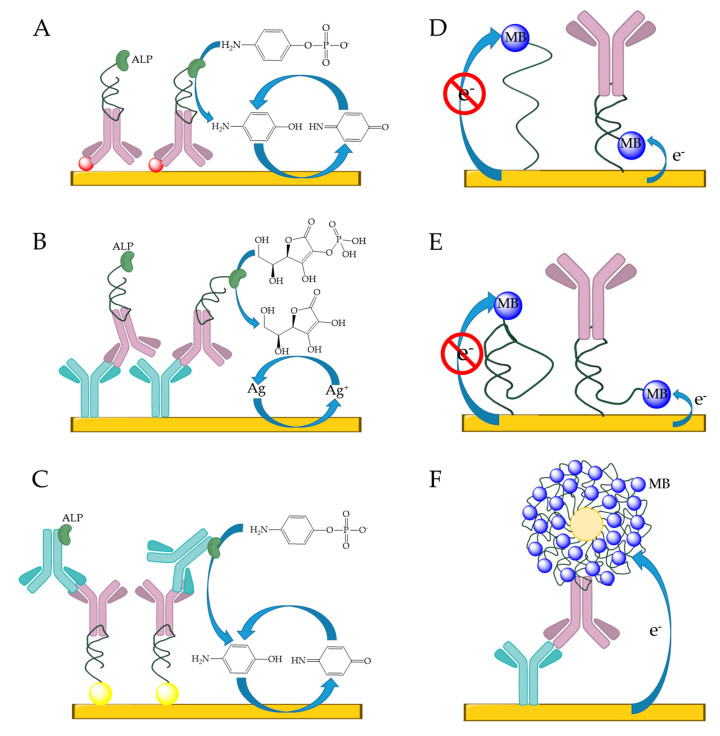
Voltammetric measurements, including: ALP activity in sandwich assays ((**A)** [151]; (**B)** [152]; and (**C)** [153]); conformational switching aptamers ((**D)** [157]; and (**E)** [158]); and methylene blue accumulation in the G-rich domain of the aptamer ((**F)** [159]).

Voltammetric measurements have widely been used for IgE by coupling with (bio)catalytic or redox labels in sandwich assays. Papamichael and coworkers developed an alkaline phosphatase enzyme (ALP)-based sandwich assay, where the antigen of IgE was immobilized onto an electrode surface followed by the binding of IgE, which enabled capping with the biotin-modified aptamer. Extravidin–alkaline phosphatase conjugate was further reacted with biotinylated aptamer, and then the IgE was quantified by enzyme hydrolyzed *p*-aminophenyl phosphate (*p*-APP) conversion to *p*-aminophenyl (*p*-AP), an electrochemically active product detected by differential pulse voltammetry (DPV) at the underlying electrode surface (Figure 10A) [151]. Similar sandwich assays were established by replacing the IgE antigen with a capture anti-IgE antibody. In this particular case, ascorbic acid 2-phosphate was used as a substrate hydrolyzed by ALP to ascorbic acid. In an attempt to increase the sensitivity, instead of detecting the ascorbic acid directly, its reducing properties were used to deposit metal silver from Ag^+^ on the electrode surface detected by stripping voltammetry (Figure 10B). The amount of the accumulated silver scaled with the IgE concentration down to an LOD of 20 pM [152]. The versatility of these type of assays is demonstrated by the possibility to modify the electrode through gold nanoparticles with the aptamer and using ALP-labeled anti-IgE antibody as tracer (Figure 10C). The nanoparticle-modified electrode setup proved to have several advantages over the more conventional approaches and ultimately led to an LOD of 5 fM [153].

An interesting example of using a self-reporting format that is apart from the majority of voltammetric methods that use a tracer affinity reagent was introduced by Li et al. An aptamer is used that is linked through a thiol label to a gold electrode while the other terminus is modified with an ALP. In the absence of IgE, the ALP converts 1-naphthyl phosphate (1-NP) to the electroactive naphthol, which is oxidized on the electrode surface. Interestingly, the IgE binding “buries” the enzyme and as such its catalytic activity is suppressed in an IgE concentration-dependent manner. Surprisingly, this relatively simple assay construct led to a remarkably good LOD of 22.7 fM [154].

Such self-reporting approaches, in which the conformational switch of the aptamer triggers a signal, are more common using redox labels (most often methylene blue (MB)) that are approached or distanced from the electrode surface upon the selective target binding and as such turn-on or turn-off the current, respectively [155]. Apparently, the native IgE aptamer is not a good candidate for such an approach, as merely 2.5% current change was detected upon IgE binding despite the large size of the protein [156]. Thereby re-engineering of the aptamer was required by the destabilization of the hairpin structure (Figure 10D) [157] or by the elongation of the DNA producing a pseudoknot aptamer probe (Figure 10E) [158]. A ca. 20% change in the current was measured by square wave voltammetry (SWV), when destabilization of the stem region was carried out with substituting a G to T at position 30 with only a slight worsening of the equilibrium dissociation constant of IgE-modified aptamer complex with respect of the original sequence [157]. The pseudoknot-type aptamer probe led to even better results and reached an LOD of 60 pM. The specific aptamer contained a two-stem-loop structure, where a domain of the loop is hybridized to form the stem domain of the other and the two ends of the aptamer were modified with thiol and methylene blue for immobilization and electrochemical detection, respectively. In the absence of IgE, the rigid pseudoknot structure was stable, holding the redox label far from the electrode surface. However, in the presence of IgE, the rigid structure was opened, and the methylene blue was brought into close proximity of the electrode surface to allow direct electron transfer, which was measured by SWV. Moreover, the sensor was able to measure IgE concentration in human serum, as well [158].

It is not required to chemically modify the aptamer with the methylene blue for electrochemical detection, since this cationic dye can bind to the G-rich sequence of the aptamer. This property of MB was used in a sandwich assay-based method, in which the anti-IgE-captured IgEs were capped with aptamer-modified gold nanoparticles. The high probe density on the gold nanoparticles led to an accumulation of the added MB that could be detected by DPV (Figure 10F). Obviously, an antibody is required as a capture probe to eliminate the background signal (MB is not able to bind to proteins). This approach claims an LOD of 0.52 ng/mL (2.7 pM) and a large dynamic range (1–10,000 ng/mL (5.3 pM–52.6 nM)) [159]. In contrast with the previous method, direct electrochemical detection could also be used, when aptamers were immobilized on carbon nanotube, ionic liquid and chitosan-modified electrode. The methylene blue was interacted with the guanine bases in the absence of the IgE producing a well-defined DPV peak. In the presence of IgE, the MB was released and the peak current was dropped. A 37 pM LOD was achieved, and the sensor was capable to detect IgE selectively even in human serum [160]. A similar “signal-off” method was used, when aptamers were immobilized on gold nanoparticles deposited on the electrode. In the absence on IgE, a long complementary DNA strand could hybridize to the aptamer where MB could incorporate into the G-rich regions detectable by SWV. In the presence of IgE, the cDNA was not able to hybridize to the aptamer strand, thereby the peak current was decreased. The sensor enabled to measure IgE from 1 pM to 100 nM with 0.16 pM LOD [161]. 

Streptavidin functionalized and AgNP decorated graphene was used as a redox labeling in square wave anodic stripping voltammetry measurements. A sandwich immunoassay was built up for selective and sensitive detection of IgE with 3.6 ng/mL (19 pM) LOD. 

On gold-coated screen-printed electrodes, thiol-modified aptamer strands were immobilized along with mercaptohexanol. Upon IgE binding, biotin-labeled anti-IgE antibody was able to hybridize followed by the binding of streptavidin-modified and AgNP-loaded graphene sheet. The signal amplification was ensured by the high loading capacity of AgNP on the graphene allowing the SWAV measurements of Ag^+^ [162].

The development of an antifouling sensing interface to allow for ultrasensitive and specific detection with voltammetry received also attention. Macroporous gold surface was prepared byelectrochemical gold deposition on multilayer polystyrene nanospheres followed by the removal of the nanospheres. Thiol-labeled aptamers and zwitterionic peptides were immobilized to prevent non-specific adsorption owing to its hydrophobicity, neutral charge and a well-defined secondary structure, which ensures closely packed monolayer. IgE prevents the electron transfer between the [Fe(CN)_6_]^3−/4−^ and the electrode, thereby the peak current in DPV was significantly dropped and 42 fg/mL (0.22 fM) detection limit was achievable [163]. The macroporous gold could be changed with poly (*m*-aminobenzoic acid) (PABA) electrochemical deposited on the electrode surface followed by immobilization of the aptamer and the zwitterionic peptide [164].

### 6.3. Electrophoretic Methods for IgE Detection

Given the large mass change upon binding IgE, capillary electrophoresis-based separation of the aptamer–IgE complex from free aptamers is readily possible. Moreover, the use of fluorescent-labeled aptamers made the detection by laser-induced fluorescence (CE-LIF) especially convenient. Such separations and detections can be carried out in ca. 1 min, with two well separated peaks corresponding to the free and IgE bound forms of fluorescent-labeled aptamer that enabled their quantitfication with five orders of magnitude dynamic range and 46 pM LOD [165]. Aspects to consider in CE-LIF are the use of appropriate labels that does not interfere with the target binding, which can be ensured by choosing an appropiate linker between the aptamer strand and the fluorophore or by changing the the position of the label. Clearly, the complex formation and the stability during separation is influenced by the buffer composition and the best sensitivity in complex detection was observed at the shortest separation time, although the increase of the electric field could cause the dissociation of the aptamer–target complex [166]. To enhance the binding affinity between the aptamer and the target molecule, a polyT extension on the 5′ end can be applied to gain well-isolated peak for the aptamer–IgE complex in CE-LIF with higher intensity at the same concentrations compared to the original method [167]. Alternative detection methods include chemiluminescence [168] or PCR [169] involving additional reagents after the separation was performed, thus less convenient than the fluorescence-based detection, but higher sensitivities while still low overall analysis times was offered, e.g., in the case of chemiluminescence detection with an analysis time of 5 min, an LOD in the fM range was achieved [168].

Microfluidic CE devices have also been fabricated since they have several advantages over conventional CE including reduced reagent necessity, minimized cost and analysis time, thereby they show excellent prospects for aptamer-based protein detection coupled with electrophoresis. Wide range of microfluidic devices were used including gel-shift detection platform incorporating competitive inhibition strategy [170], DNA mobility shift assay [171] and frontal analysis [172]. Since the aptamer–IgE complex is difficult to measure with conventional methods—due the slow migration of the complex—free flow electrophoresis (FFE) was applied with fast measurements and accurate results [173,174], i.e., in FFE, the sample is progressed by pressure driven flow and the electrical field applied perpendicularly to the direction of the flow only deviates the various components based on their mass/charge ratio from their linear trajectory. Perhaps, the best analytical performance has been achieved with a homogeneous mobility shift assay employing a complex amplification mechanism. The relatively low affinity of aptamer–IgE complexes that could lead to their dissociation during separation was circumvented by continuous sample injection to shift the equilibrium towards complex formation coupled with an electrokinetic concentration for a continuous signal amplification and to minimize band dispersion by self-focusing. The lab-on-chip device was able to detect 4.4 and 39 pM IgE in buffer and 10% serum, respectively [175].

## 7. Conclusions and Outlook

In this review, the current state of the art in immunoglobulin specific aptamer-based biosensors is compiled. The main uses of the aptamers selected for immunoglobulins are very broad and include: (i) affinity purification; (ii) reporting molecules replacing secondary antibodies; and (iii) selective capturing or detecting reagents. In terms of affinity purification, their high, but easily controllable, affinity makes them suited for the purification of antibodies under mild conditions to preserve the activity of the antibodies.

This compilation shows a clear dominance of the aptamer-based detection of IgE as compared with other immunoglobulin targets. This is by far not justified by the diagnostic importance of IgE as compared with other immunoglobulins, but, given the excellent affinity and selectivity of IgE aptamers, by their use as a convenient model to develop new detection concepts and analytical platforms. Many of these detection concepts exploit the intrinsic properties of the nucleic acid aptamers that show appealing advantages and versatility compared to antibody-based reagents and as such they might provide a useful snapshot on the general possibilities in this field. Particularly, the amenability of aptamer reagents for PCR amplification and their negative charge opened new detection routes hardly possible by conventional immunoassays. While aptamers in the present report routinely enabled subnanomolar LODs, the innovative use of their properties led to LODs in the fM range.

Despite all their claimed advantages, the use of aptamers as diagnostic reagents for antibody determinations still strikingly lags behind that of the conventionally used reagents. Presently, the standard diagnostic methodologies targeting immunoglobulin biomarkers are largely based on antibodies. Thus, infection-generated IgG and IgM are detected from serum and plasma samples by sandwich ELISA or lateral flow immunoassays; the detection of the extraordinary IgG antibody expression caused by cancer and autoimmune disease is determined by sandwich immunoassays [176]; and conventional determinations of IgE also employ antibody-based sandwich ELISA, RAST (Radio Allergo-Sorbent Test in serum) or immunoblot tests [51].

Indeed, aptamers, beside their many advantages, also have some drawbacks starting from the inherently probabilistic nature of the selection processes, which cannot guarantee that sufficiently high affinity aptamers, fully lacking of cross-reactivity to coexistent species in complex sample matrices, such as whole blood, serum, saliva, etc., can be delivered. The large negative charge density and lack of hydrophobic moieties in the case of natural nucleic acid aptamers are also rather prohibitive in recognizing either negatively charged or hydrophobic immunoglobulin epitopes. In terms of integration in sensor platforms, as their structure is more flexible and as such susceptible to environmental conditions (pH, ionic strength, degradation by nucleases), the immobilization, chemical modification and the medium in which the assay is performed need more consideration than in the case of antibody-based reagents.

However, the advances in the field have largely addressed these drawbacks. Most importantly, the implementation of non-natural nucleic acids diversified the range of non-covalent interactions, increased the success rate of the selection (making it also less dependent on the target) and provided nuclease resistance. The IgA aptamer presented is a relevant example in this respect. The counter-selection emerged also as a powerful tool to custom adjust the selectivity of the aptamers for the application, by removing those sequences that bind to critical interferences and matrix components.

Overall, the number of methodologies for selection, control and screening seems up to the challenge of generating aptamer-based diagnostic reagents for antibodies, admittedly at the expense of a more complex and costly selection process. However, this is perhaps a small price to pay given the benefits in terms of their chemical synthesis (solely based on the knowledge of the nt sequence), accordingly batch-to-batch consistency, excellent temperature and biochemical stability ideal for integration in long shelf-live cartridges, and the overall versatility of nucleic acid chemistry in terms of controlled labeling.

## Figures and Tables

**Table 2:** Selected aptamers against SIgA and their equilibrium dissociation constants.

Name	Sequence (5′-3′) ^2^	K_D_ (nM)	Ref.
**IgA^gu1^**	GGT TTG GAC GCA ATC TCC CTA ATC xGC xGA xGx xxG xAx xxC AAA xxA GCC GCA GAA ACx ACA AxG GG CGG GCx xAx C	13.6	[16]
**IgA^gu1-3^**	GCA ATC TCC CTA ATC xGC xGA xGx xxG xAx xxC AAA xxA GC	3.7	
**IgA^gu1-3-3^**	AAT CTC CCT AAT CxG CxG AxG xxx GxA xxx CAA Axx	10.4	

^2^ x letters indicate the U^gu^ bases.

**Table 3:** Selected aptamers against IgMs and their equilibrium dissociation constants at 37 °C and 4 °C.

Name	Sequence (5′-3′)	K_D_ (nM)	Ref.
**TD05**	AAC ACC GTG GAG GAT AGT TCG GTG GCT GTT CAG GGT CTC CTC CCG GTG	>10,000 359^3^	[25,55]
**TD05.1**	AGG AGG ATA GTT CGG TGG CTG TTC AGG GTC TCC TCC T	>10,000 53^3^	[55]
**TD05.17**	AGG AGG ATA GTT CGG TGG CTG TTC AGG GTC TCC TCC T ^4^	>10,000 43^3^	
**R1**	ATC CAG AGT GAC GCA GCA AAC ACT GGG TGG GGT TAG CGG GCG ATT TAG GGA TCT TGA GTG GTG GAC ACG GTG GCT TAG T	315^3^	[26,60]
**R1.1**	CAC TGG GTG GGG TTA GCG GGC GAT TTA GGG ATC TTG AGT GGT GGA CAC GGT GGC TTA GT	73.2^3^	[60]
**R1.2**	CAC TGG GTG GGG TTA GCG GGC GAT TTA GGG ATC TTG AGT GGT	35.5^3^	

^3^ K_D_ values at 4 °C. ^4^ The underline letters indicate the locked nucleic acid bases.

**Table 4:** IgG Fc domain specific aptamers with their sequences and K_D_ values.

Species	Name	Sequence (5′-3′) ^5,6^	K_D_ (nM)	Ref.
**human**	Apt8-2	GGA GGU GCU CCG AAA GGA ACU CC	117	[17]
	Apt131	**GGA**GGU GC**U C**C**G AAA**GG**A** ACU **CC**	NA	[66]
**rabbit**	R18	GGG AGA AUU CCG ACC AGA AGU UCG AUA CGC CGU GGG GUG ACG UUG GCU ACC CUU UCC UCU CUC CUC CUU CUU CU	0.015	[6]
**mouse**	-	TAA TAC GAC TCA CTA TAG CAA TGG TAC GGT ACT TCC AAG CTA ACC CTC ATC TGC GCG CTC CCA AAA GTG CAC GCT ACT TTG CTA A	NA	[11]
	6	TAA TAC GAC TCA CTA TAG CAA TGG TAC GGT ACT TCC CCA CTC ACC GGG TAC CTG CCG CTC CCA AAA GTG CAC GCT ACT TTG CTA A	NA	[5]
	12	TAA TAC GAC TCA CTA TAG CAA TGG TAC GGT ACT TCC AAG CTA ACC CTC ATC TGC GCG CTC CCA AAA GTG CAC GCT ACT TTG CTA A	NA	

^5^ The 2′-fluoro nucleotides are underlined. ^6^ The 2′-O-methyl modifications are indicated with bold letters.

**Table 6:** Aptamers against different target autoantibodies, their chemical modification and K_D_ values.

Autoimmune Disease	Target of Autoantibody	Target of Aptamer	Chemistry	K_D_ (nM)	Ref.
**MS**	myelin basic protein (MBP)	polyclonal anti-MBP	2′-F-Py RNA	1.2	[13]
			2′-F-Py RNA	3	[14]
**SLE**	DNA	monoclonal G6-9 anti-DNA AAB	RNA	2	[81]
**DCM, ChCM, PPCM**	beta1-adrenoceptor	β1-ECII-AAB	ssDNA	NA	[82,83]
			ssDNA, truncated	NA	[84,85]
	G-protein coupled receptor	GPCR-AAB	SsDNA ^7^	NA	[86,87,88,89]
**T2DM**	human insulin receptor on lymphocytes	mouse monoclonal antibody (MA20)	RNA	2	[90]
			2′-A-Py RNA	30	[91]
**MG**	acetylcholine receptor (AChR)	rat monoclonal antibody (mAb198)	2′-A-Py RNA	60	[92]
			2′-F-Py RNA	25	[93]
			2′-A-Py RNA, elongated at 3′ end	6	[94]
			2′-F-Py RNA	30	[95]
			2′-F-Py RNA, PEG at 5′ end	75	[95]
			2′-A-Py RNA, truncated	NA	[96]

^7^ Aptamer, which was originally selected for thrombin (ARC 183 vs. BC 007).

**Table 7:** Aptamers against antitoxoplasma, used in sandwich assay.

Target	Name	Sequence (5′-3′)	K_D_ (nM)	Ref.
**antitoxoplasma**	TGA6	GGG AGC TCA GAA TAA ACG CTC AAC GCA TTT CGC AAC ACG GCT TGG CAA CGT TCC TGG TTC GAC ATG CGG CCC GGA TC	NA	[15]
	TGA7	GGG AGC TCA GAA TAA ACG CTC AAC GCA TTT CGC AAC ATG ACT TCC CAA CGT TCC TGG TTC GAC ATG CGG CCC GGA TC	NA	

**Table 8:** The most widely used IgE selective aptamers.

Name	Type	Sequence (5′-3′)	K_D_ (nM)	Ref.
**IGE1.2**	RNA	GGG AGG ACG AUG CGG GUG UGA AUG GUG UUG UGA GG	30	[7]
**D17.4**	DNA	GGG GCA CGT TTA TCC GTC CCT CCT AGT GGC GTG CCC C	10	

**Table 9:** Parameters of SPR measurements of D17.4 aptamer–IgE interaction.

Immobilization	Sequence of DNA ^8^	Working Buffer	K_D_ (nM)	Ref.
EDC/NHS Streptavidin–biotin	5′-**GGG GCA CGT TTA TCC GTC CCT CCT AGT GGC GTG CCC C** -T_24_-biotin-3′	TGK	30.9	[110]
EDC/NHS Streptavidin–biotin	5′-GCG C**GG GGC ACG TTT ATC CGT CCC TCC TAG TGG CGT GCC CC** GC GC-biotin-3′	HBS	NA	[109]
Au-S	5′-thiol-AAA AAA AAA A**GG GGC ACG TTT ATC CGT CCC TCC TAG TGG CGT GCC CC**-3′	PBS	74.6	[111]
EDC/NHS	5′- NH_2_-ethylene-glycol - **GGG GCA CGT TTA TCC GTC CCT CCT AGT GGC GTG CCC C**-3′	TGK	54.3	[107]
Thiol	5′-**GGG GCA CGT TTA TCC GTC CCT CCT AGT GGC GTG CCC C**TT TTT TT-(CH_2_)_6_-SH-3′	PBS, 1 mM MgCl_2_	0.73	[112]
EDC/NHS (Immobilization of IgE)	5′-**GGG GCA CGT TTA TCC GTC CCT CCT AGT GGC GTG CCC C**-3′	PBS, 1 mM MgCl_2_	4.7	[108]
EDC/NHS Streptavidin–biotin	5′-**GGG GCA CGT TTA TCC GTC CCT C CTA GTG GCG TGC CC**-T24-biotin-3′	HBS-EP	NA	[116]
Au-S	5′-SH-AAA AAA AAA AAA AAA **GGG GCA CGT TTA TCC GTC CCT CCT AGT GGC GTG CCC** G-3′	PBS	4.55	[113]
Au-S	5′-SH-**GGG GCA CGT TTA TCC GTC CCT CCT AGT GGC GTG CCC C**-3′	PBS	270	[114]
Au-S	5′-**GGG GCA CGT TTA TCC GTC CCT CCT AGT GGC GTG CCC C**TT TTT -(CH_2_)_3_-SH-3′	PBS, 1 mM MgCl_2_	0.32 (0.94 in PBS)	[115]

^8^ The **bold** letters indicate the original sequence, which was selected by Wiegand et al. [7].

**Table 10:** Analytical parameters of heterogeneous fluorescent assays for IgE determination.

Detection Principle	Linear Range (pm)	LOD (pM)	Ref.
Aptamer-modified poly(di-acetylene) supramolecules (Figure 8A)	5.3–5260	0.53	[121]
Fluorescence enhancement by silver nanoparticle hybrid probes (Figure 8B)	2.6–5260	0.21	[122]
Metal-enhanced fluorescence with silver nanostructures (Figure 8C)	2.6–84	1.4	[123]
Metal-enhanced fluorescent probes based on silver nanoparticles (Figure 8D)	53–32,900	5.3	[124]
Paper-supported aptasensor based on luminescence resonance energy transfer using upconverting nanoparticles (Figure 8E)	2.6–420	NA	[125]
Non-stripping gold nanoparticles triggered chemiluminescence (Figure 8F)	0.2–1000	0.05	[126]
Fluorescence quenching by graphene oxide	60–225	22	[127]
